# Supplementary material for: Analysis of the deterioration process of DC XLPE cable with protrusion defect based on the development of partial discharge
Source: PLoS One. 2025 Jun 25;20(6):e0326271. doi: 10.1371/journal.pone.0326271 (PMC12192135; doi:10.1371/journal.pone.0326271)
Supplement: Attachment — Submitted filename: Manuscript (2).docx [file pone.0326271.s001.docx]

# Analysis of the deterioration process of DC XLPE cable with protrusion defect based on the development of partial discharge

Yunjie Zhou ^1^, Shuting Yang ^1^, Jiamin Xu ^1^, Haosheng Lv ^1^, Jialiang Yuan ^1^and Baiyu Li ^2,^*

^1^	State Grid Shanghai Cable Company, Shanghai 200072, China, [zyj13601620501@163.com](mailto:zyj13601620501@163.com)(Y.Z); 1430143791@qq.com(S.Y); merriline@126.com(J.X); xiaolu0008@126.com(H.L); yjl15026571090@163.com(J.Y)

^2^	Shanghai Jiao Tong University, Shanghai 200240, China, z1mubaiyu@sjtu.edu.cn

*****	Corresponding author E-mail: [z1mubaiyu@sjtu.edu.cn](mailto:z1mubaiyu@sjtu.edu.cn)

# Abstract

Protrusion defect is easily introduced in the cross-linked polyethylene (XLPE) high voltage direct current (HVDC) cable manufacturing process. It will distort the electric field and cause partial discharge (PD). Therefore, partial discharge experiments are carried out at 50 ℃ and 80 kV for internal semi-conductive protrusion defects in insulation in this paper. The whole discharge process is divided into discharge stages, and the changing trend of average discharge frequency, average discharge quantity, cumulative discharge quantity, and their change rate are analyzed in stages to further explore the defect development mechanism behind the evolution of discharge stages of the defect. The experimental results show that the average discharge frequency and average discharge quantity both show an obvious trend of continuous increase. The cumulative discharge has an obvious rapid rising trend, especially in the fourth stage of discharge. At the same time, the simulation shows that the extension of the discharge channel is closely related to the increase in discharge quantity. This indicates that the protrusion defect develops to a greater extent during the whole voltage application process, especially during the fourth stage of discharge.

# Keywords:

HVDC cable, XLPE insulation, Protrusion defect, Partial discharge, Insulation deterioration.

# 1. Introduction

At present, with the development of new energy technologies such as photovoltaic power generation [1,2], renewable energy has not only been more applied in life [3-6], but also participated in more grid-connected operations [7]. This makes the flexible DC transmission technology that can effectively connect new energy to the main network get attention. With the improvement of the voltage level of flexible HVDC lines, the safety of power equipment such as high voltage transformers [8] and HVDC cable has received more and more attention. As an important equipment in a flexible DC transmission system, DC cable will fail due to internal aging. To reduce the aging fault in the cable, on the one hand, the insulation performance can be improved at the material level by developing new insulation materials [9,10], or the modification treatment method [11-13] of commonly used insulation materials such as nano-doping. On the other hand, the state of the cable can be evaluated by studying the physical phenomena before the aging fault occurs. Partial discharge is a common physical phenomenon associated with the aging of high voltage cables.

The appearance of partial discharge is usually due to the defects introduced in the cable insulation, and the continuous partial discharge accelerates the insulation aging and seriously affects the insulation performance of the cable [14,15]. Under continuous discharge, the defects continue to develop until a distinct slender discharge channel is formed [16]. The arborescent discharge channel will continue to grow in the more intense discharge, when the field strength within the remaining insulation layer thickness is higher than the minimum breakdown field strength, the electrical branch will rapidly develop through the entire insulation, and the insulation layer is broken down. Therefore, it is necessary to study the discharge channels and discharge characteristics caused by defects in cables.

However, most of the studies on the electrical branches and their discharge under DC voltage are usually carried out with the pin-plate electrode based on the XLPE sample [17,18]. The development of protrusion defects based on actual XLPE cables and their discharge studies are rarely involved. The discharge channel cannot be directly observed in the cable, but its partial discharge characteristics have a strong correlation with the development of the discharge channel [19]. In this paper, a constant voltage is applied to the protrusion defect in DC cable at a certain temperature to collect the change of partial discharge characteristics. The geometric model of the cable protrusion defect is established in the finite element simulation software, and the electric field simulation and partial discharge model based on electron avalanche are developed. The relationship between the degree of electric field distortion at the defect and the discharge quantity and the size of discharge channel is investigated by the model. The development of the protrusion defect in different stages can be deduced by dividing the stages by the variation of discharge characteristics. This paper can provide a favorable reference for the evaluation of XLPE cable insulation status by partial discharge.

# 2. Experimental and simulation methods

## 2.1. Sample preparation

This paper selects the use of 8.7/15 kV single-core cross-linked polyethylene cables in experiments. These cables are chosen to study the partial discharge characteristics during their evolution. The cable's insulation is identified as LS4258DCE from Borealis and the shielding layer is LE0550DC, which is compatible with the insulation. The primary parameters are detailed in Table 1.

Table 1. XLPE cable specification used to test.

| Structure | Parameters | Material/Process |
| --- | --- | --- |
| Nominal Cross-Section | 95mm^2^ | / |
| Conductor Outer Diameter | 11.5mm | Copper Wire Stranding |
| Inner Shielding Thickness | 0.8mm | Borealis LE0550DC |
| Insulation Thickness | 4.5mm | Borealis LS4258DCE |
| Outer Shielding Thickness | 1.2mm | Borealis LE0550DC |
| Sheath Thickness | 2mm | Semiconductive Polyethylene |

The cable comprises a 6-layer structure, including a copper core, an inner semi-conductive shield, XLPE insulation, an outer semi-conductive shield, a copper tape shield, and an outer sheath. Protrusion defect is made as a cone protruding from the inner semi-conductive shielding layer towards the insulation, with a height of 1 mm and a base radius of 0.2 mm, just as in Fig 1. The material of the cone is the same as that of the inner semi-conductive shielding layer. The specific production process is as follows:

1) Cut off the outer semi-conductive layer, the insulating layer and the inner semi-conductive layer successively in the middle of the cable.

2) Then restore the inner semi-conductive layer: After vulcanization, the thickness of the restored inner semi-conductive layer is about 2 mm larger than that of the inner semi-conductive layer.

3) Cut a 1mm protrusion in the middle of the restored inner semi-conductive layer, and scrape away the excess semi-conductive layer in the rest, that is, form the inner semi-conductive layer "protrusion".

Fig 1. Profile of protrusion defect in the cable.

As shown in Fig 2, the total length of the cable is 45 cm, with the defect located at the midpoint. To avoid surface tracking and corona discharge, 7 cm of the outer semi-conductive shielding layer at both ends of the cable was stripped to expose the XLPE insulation. Subsequently, 3 cm of the XLPE insulation at each end was removed to expose the conductor, facilitating the insertion of a grading ring and direct connection of the high-voltage end to the conductor. All surfaces were maintained smooth. At the central position of the cable, a 3-5 cm wide section of the outer sheath was removed to expose the metallic shielding layer, facilitating the connection of the grounding wire. The entire cable and its connecting parts are soaked in insulating oil to avoid corona.

|  |  |
| --- | --- |
| (a) schematics. | (b) Physical drawing. |

Fig 2. Wiring diagram of the cable.

## 2.2. Experimental circuits and methods

The high voltage circuit and partial discharge detection system for the DC cable are illustrated in Fig 3. The DC high voltage source is a model ZLT-**±**1200/50. The AC voltage is rectified by a high-voltage silicon stack, filtered through a 0.1-μF stabilizing capacitor to obtain DC voltage, and connected to a 100-kΩ protective resistor to prevent high currents in the event of cable breakdown. The capacitance value of the coupling capacitor is 1000 pF. To eliminate discharges from the experimental system itself, all high-voltage circuits are equipped with voltage-equalizing hoods, and the possibility of corona discharge is excluded.

Fig 3. The test circuit of DC cable partial discharge.

Synchronous measurements of partial discharge in cables were conducted using the impulse current method, with a measurement frequency range of 10 kHz to 1 MHz. In this study, discharges were characterized in terms of discharge quantity, measured in pico coulombs (pC), and corrected using a square wave correction module. Background noise for cables is tested under an 80-kV DC voltage condition, without artificially induced defects, did not exceed 10 pC.

Considering that the discharge characteristics during the degradation process of defects involve a continuous evolution, an extended duration of local discharge experiments was conducted using a cyclic oil bath heating method in this study. Due to the small size of defects and their relatively mild impact on insulation in this study, it is challenging for defects to undergo significant development and discharge evolution at room temperature. Excessive temperatures can accelerate defect degradation to the point of breakdown, leading to rapid changes in discharge characteristics. In this paper, a cyclic oil bath heating system is employed to heat the DC cable samples, with the cable temperature maintained at 50 °C throughout the process.

It is important to note that this temperature does not represent the full range of operating conditions for HVDC cables. In fact, the working temperature of HVDC XLPE cable is greatly affected by the laying environment, external temperature, and current, and the long-term working temperature from normal temperature to 70 degrees Celsius is reasonable.

The constant voltage method is used to apply voltage in this paper. To observe the evolution of discharge characteristics of typical defects, it is crucial to select an appropriate applied voltage. The excessive applied voltage can lead to rapid defect development and breakdown, while too low a voltage may result in slow defect evolution, leading to excessively long experimental durations. After preliminary experiments, the experimental voltage for typical defects is set at 80 kV.

To ensure the rigor of the experiment, the experiment was carried out three times. Due to the limitation of cable technology and the randomness of partial discharge, the data of each experiment could not be the same, but the range and variation trend of discharge parameters were consistent. After collecting all the discharge waveforms of the pressure process through the oscilloscope, they are stored in the form of “txt” to the computer for processing. Because the source of abnormal data can only be electromagnetic interference, the O-coupled signal frequency and amplitude of electromagnetic interference (<10 pC) and discharge are very different. After the abnormal data are eliminated, the experimental data are sorted out, mapped, and further analysed.

## 2.3. Electric field simulation

To measure the degree of electric field distortion of protrusion defects, the defects are modeled geometrically in finite element software, and the electric field distribution is solved by an electrostatic field module. The mesh needs to be divided into smaller parts to correctly calculate because the radius of curvature at the protrusion is small. The mesh size at the protrusion is set to 2 μm. The conductor voltage is set to 80 kV. The length of the protrusion defect is 1 mm.

The only material parameters required for the physical field are relative dielectric parameters. Here, the relative dielectric constant of the semi-conductive layer is set to 20. The relative dielectric parameter of XLPE is 2.3. The relative dielectric constant of air is 1. On the boundary conditions, only the conductor voltage needs to be set to 80 kV. The outer semi-conductive layer of the cable should be connected to the ground. The steady-state study was selected.

## 2.4. Partial Discharge Simulation

Although we have understood the degree of electric field distortion at the defect, it is the partial discharge at the defect that really damages the insulation material. Partial discharge is a complex and constantly changing physical process. To explore what exactly happens at the microscopic level of partial discharge of the protrusion defect, the finite element simulation software was used to establish a plasma model under multiple physical fields, and the charge migration and diffusion, collision ionization, and composite adhesion behaviors were analysed during the discharge process.

There is a set of hydrodynamic gas equations to describe the partial discharge process of the air gap mode [20]. This gas model has been widely used to simulate the microscopic simulation of closed gas discharge and has been proven to be effective.

$$\frac{\text{∂}\text{n}_{\text{e}}}{\text{∂}\text{t}}\text{+}\text{∇}\text{∙}\left（ \text{μ}_{\text{e}}\vec{\text{E}}\text{ * }\text{n}_{\text{e}}\text{-}\text{D}_{\text{e}}\text{∇}\text{n}_{\text{e}} \right）\text{ =}\text{ R}_{\text{e}}\text{ } \text{ }\text{(}\text{1}\text{)}$$

$$\frac{\text{∂}\text{n}_{\text{p}}}{\text{∂}\text{t}}\text{+}\text{∇}\text{∙}\left（ \text{μ}_{\text{p}}\vec{\text{E}}\text{ * }\text{n}_{\text{p}}\text{-}\text{D}_{\text{p}}\text{∇}\text{n}_{\text{p}} \right）\text{ = }\text{R}_{\text{p}} \text{ }\text{(}\text{2}\text{)}$$

$$\frac{\text{∂}\text{n}_{\text{n}}}{\text{∂}\text{t}}\text{+}\text{∇}\text{∙}\left（ \text{μ}_{\text{n}}\vec{\text{E}}\text{ * }\text{n}_{\text{n}}\text{-}\text{D}_{\text{n}}\text{∇}\text{n}_{\text{n}} \right）\text{ =}\text{ R}_{\text{n}}\text{ } \text{(}\text{3}\text{)}$$

$$\text{∇}^{\text{2}}\text{V = }\frac{\text{-e}\text{(}\text{n}_{\text{p}}\text{-}\text{n}_{\text{e}}\text{-}\text{n}_{\text{n}}\text{)}}{\text{ε}_{\text{r}}\text{*}\text{ε}_{\text{0}}} \text{(}\text{4}\text{)}$$

where *n_e_*, *n_p_*, and *n_n_* are the charge body densities of electrons and positive and negative ions. *μ_e_*, *μ_p_*, and *μ_n_* are the mobility of electrons and positive and negative ions under an electric field. $\vec{\text{E}}$ is the electric field intensity vector. *D_e_, D_p_*_, and_ *D_n_* are the diffusion coefficients of electrons and positive and negative ions driven by the concentration gradient. *R_e_, R_p_*, and *R_n_* are the source terms of electrons and positive and negative ions, and *V* is the electric potential. 𝜀*_r_* is the relative dielectric constant; 𝜀_0_ is the dielectric constant of vacuum; 𝑒 is the charge on an electron.

Equations (1~3) show the variation process of the spatial concentration of charged particles. Taking Formula (2) as an example, it is explained in detail that $\frac{\text{∂}\text{n}_{\text{p}}}{\text{∂}\text{t}}$ represents the concentration change of positive ions over time, $\text{∇}\text{∙}\left（ \text{μ}_{\text{p}}\vec{\text{E}}\text{ * }\text{n}_{\text{p}}\text{-}\text{D}_{\text{p}}\text{∇}\text{n}_{\text{p}} \right）$ represents the change of positive ions due to migration and diffusion, and$\text{ }\text{R}_{\text{p}}$ is the difference between the rate of positive ions generation and disappearance. In addition, considering that the migration and dissipation of charged particles can lead to changes in the electric field, this is combined with Poisson's equation (4).

$$\text{R}_{\text{e}}\text{ =}\text{ }\left| \text{μ}_{\text{e}}\vec{\text{E}} \right|\text{ * }\text{n}_{\text{e}}\text{*}\text{α-}\left| \text{μ}_{\text{e}}\vec{\text{E}} \right|\text{ * }\text{n}_{\text{e}}\text{*}\text{η-}\text{β}_{\text{ep}}\text{n}_{\text{e}}\text{n}_{\text{p}} \text{ (5)}$$

$$\text{R}_{\text{p}}\text{ = }\left| \text{μ}_{\text{e}}\vec{\text{E}} \right|\text{ * }\text{n}_{\text{e}}\text{*α-}\text{β}_{\text{np}}\text{n}_{\text{n}}\text{n}_{\text{p}}\text{-}\text{β}_{\text{ep}}\text{n}_{\text{e}}\text{n}_{\text{p}} \text{(6)}$$

$$\text{R}_{\text{n}}\text{ =}\left| \text{μ}_{\text{e}}\vec{\text{E}} \right|\text{ * }\text{n}_{\text{e}}\text{*η-}\text{β}_{\text{np}}\text{n}_{\text{n}}\text{n}_{\text{p}}\text{ } \text{(7)}$$

𝛼 is the ionization coefficient, where the impact ionization coefficient is taken; 𝜂 is the adsorption coefficient; $\text{β}_{\text{ep}}$ and$\text{β}_{\text{np}}$ are the recombination coefficients of electron and positive ion and positive and negative ion respectively.

The generation and disappearance of charged particles are described by the equation of source term (5~7). In the above equation, the specific values of the above simulation parameters were obtained from the literature [21,22].

|  |  |
| --- | --- |
| (a) Geometric structure | (b) mesh generation |

Fig 4. Schematic diagram of discharge simulation and grid division.

The schematic diagram of the two-dimensional geometric model is shown in Fig 4 (A), and the geometric dimensions are consistent with those in Table 1. Free triangular mesh is selected for grid division because the time scale of gas discharge migration is small and it is greatly affected by an electric field, it needs to be divided very finely to ensure that the charge migration process of the simulation process can be accurately completed. The defect cavity was calibrated to a hydrodynamic partition, the mesh size was "extremely fine", and the maximum cell size was set to 2 μm, containing a total of 48882 in-domain cells. The division diagram is shown in Fig 4 (B).

In the simulation, to compare the microscopic discharge process in discharge channels of different lengths, the length of discharge channels is 0.5 mm and 1 mm, and the rated conductor voltage is 15 kV. Because the time scale of charged particles migration in gas discharge process is very small, the charge injection and withdrawal at gas-solid interface can be ignored in this process. In the setting of initial conditions, this paper uses a two-dimensional Gaussian distribution array to model the initial particles (electrons and positive ions) [23]. It is expressed as follows:

$$\text{n}_{\text{m}}\text{ =}\text{ N}_{\text{m}}\text{*}\text{e}^{\frac{\left（ \text{X-X}\text{0} \right）^{\text{2}}\text{+}\left（ \text{Y-Y}\text{0} \right）^{\text{2}}}{\text{-2}\text{σ}^{\text{2}}}} \text{(8)}$$

where $\text{n}_{\text{m}}$ represents the concentration distribution of the initial charged particles, and $\text{ N}_{\text{m}}$ is the peak concentration of charged particles, *X*_0_ and *Y*_0_ are the horizontal and vertical coordinates of the generated initially charged particles, and *σ* is the standard deviation and determines the rate at which the initial particles concentration decays with distance.

# 3. Results and Analysis

## 3.1. Experimental results and analysis

Take the discharge characteristics analysis of a representative experiment involving a protrusion defect in the inner shielding layer under positive polarity voltage as an example.

Fig 5. The discharge pattern of the whole experiment process of the protrusion defect.

The entire discharge process is illustrated in the discharge spectrum shown in Fig 5. The discharge duration is divided into four stages: the first stage occurs during the discharge time from 0 to 40 minutes; the second stage takes place during the voltage application time from 41 to 110 minutes; the third stage occurs during the voltage application time from 111 to 140 minutes, and the fourth stage transpires during the voltage application time from 141 to 156 minutes. The basis of our discharge stage division is a sudden change in the trend of discharge frequency and discharge quantity.

As shown in the discharge spectrum of the entire experimental process with a protrusion defect in Fig 5, during the voltage application process, the distribution range of discharge quantities falls between 10 and 300 pC, with the maximum discharge quantity even reaching above 500 pC. The overall discharge quantity exhibits a clear upward trend.

Fig 6. The average discharge of the whole experiment process of the protrusion defect.

The average discharge quantity for the entire process of the protrusion defect experiment is illustrated in Fig 6, while the discharge frequency for the entire process is depicted in Fig 7.

Fig 7. Discharge frequency of the whole experiment process of the protrusion defect.

The average discharge quantity is the arithmetic average of the apparent discharge quantity collected per minute. The discharge frequency is the number of discharges collected per minute. In the first stage of discharge, the overall discharge frequency is relatively low, approximately 60 events per minute, with minimal dispersion. The average discharge quantity per minute shows slight fluctuations, mostly ranging between 30 and 50 pC. In the second stage of discharge, the discharge frequency starts to increase, reaching up to 200 events per minute. Simultaneously, the average discharge quantity of local discharges further rises from around 50 pC at 40 minutes to approximately 80 pC at 110 minutes. In the third stage of discharge, the discharge frequency slightly increases to about 300 events per minute, and the average discharge quantity continues to rise to 100 pC. In the fourth stage of discharge, after a sudden decrease in discharge frequency for several minutes, the discharge frequency continues to increase to 600-1000 events per minute. During this process, the average discharge quantity further rises to around 120 pC, more than three times the initial discharge quantity.

|  |  |
| --- | --- |
| (a) The cumulative discharge of the experiment process | (b) The growth rate of cumulative discharge of the whole experiment process |

Fig 8. The cumulative discharge quantity and its growth rate of the whole experiment process of the protrusion defect.

It is worth noting that at 127 minutes, which is also the late third stage of discharge, the discharge frequency reaches a maximum of 1902 times per minute during the voltage application process. This is because, with the increase of voltage withstand time, the discharge frequency shows an upward trend in general. However, in the fourth stage just before the defect breakdown, the rapid development of the discharge channel leads to a decrease of the charge space density, which makes it difficult to achieve the condition of electron collapse, and the discharge frequency will decrease. Therefore, the maximum discharge frequency will appear in the middle and late 127 minutes of the third discharge stage. Surprisingly, the average discharge quantity decreases from the previous level of around 100 pC to 50 pC. This might be attributed to the presence of small-amplitude, densely occurring discharges, which have a slight lowering effect on the average discharge quantity.

Further analysis of the cumulative discharge quantity and its growth rate over time during the discharge process of the protrusion defect is depicted in Fig 8.

In the first stage of discharge, the cumulative discharge quantity exhibits slow growth, with the growth rate at a relatively low level, indicating a limited injection of discharge energy. As the discharge progresses into the second stage, both the cumulative discharge quantity and its growth rate gradually increase, reaching 1000 nC at 110 minutes. Discharge-induced damage to the solid dielectric around the protrusion defect begins to accumulate. In the third stage of discharge, the cumulative discharge quantity further increases to nearly 2000 nC, and simultaneously, the growth rate of the cumulative discharge quantity peaks, corresponding to the peak in discharge frequency at 127 minutes. This may lead to the further development of the defect and additional degradation of the insulation. In the fourth stage of discharge, the cumulative discharge quantity sharply increases, along with its growth rate, reaching 70000 pC/min. This indicates a great increase in the energy released by the insulation.

## 3.2. Simulation results and analysis

Fig 9 shows the overall distribution of the electric field in the cross-section direction from the inner semi-conductive layer to the raised protrusion defect of the cable insulation layer.

|  |  |
| --- | --- |
| (a) Electric field distribution of protrusion defect with radius of curvature of 50 μm | (b) Electric field distribution of protrusion defect with radius of curvature of 10 μm |

Fig 9. The cumulative discharge quantity and its growth rate of the whole experiment process of the protrusion defect.

The curvature radius at the tip will affect the degree of electric field distortion. Here the radius of curvature is set for two cases, 50 μm and 10 μm respectively. It can be seen that the electric field strength in most areas of the cable insulation layer is below 30 kV/mm. However, the insulation near the tip has a very serious electric field distortion. It reaches 131 kV/mm at a radius of curvature of 50 μm. When the radius of curvature is reduced to 10 μm, it can reach nearly twice the radius of curvature of 50 μm, which is 256 kV/mm.

Fig 10 shows the radial electric field intensity distribution through the center of the bottom circle of the protrusion defect. The electric field intensity rapidly declines after reaching its maximum value at the tip and gradually becomes stable at the insulation layer near the outer semi-conductive layer. It is worth mentioning that the reduction of curvature radius not only affects the electric field intensity at the tip, but also increases the electric field strength of the insulating layer near the semi-conductive layer. In general, the curvature radius will seriously affect the degree of electric field distortion.

|  |  |
| --- | --- |
| (a) 50 μm | (b) 10 μm |

Fig 10. Radial electric field intensity of protrusion defect with a curvature radius of 50 μm and 10 μm.

Through the simulation of the charge in the discharge channel, the changes in the migration and diffusion processes of electrons during a single local discharge are illustrated in Fig 11. The discharge channel has a width of 150 μm and a length of 500 μm, as shown in the fig. Following the initiation of the discharge, the migration and diffusion processes of electrons, coupled with the combination with positive ions, constitute the predominant part of the reaction. They progress toward the direction of high electric field intensity. At 1 ns, with the development of electron avalanche, the electron density at the head has reached nearly 60 times that at the beginning. By 2.1 ns, the head of the electron avalanche has almost reached the surface of the dielectric. Electrons undergo inelastic collisions with the dielectric surface, and their energy is insufficient to continue collision ionization. At 2.5 ns, the process of electron avalanche essentially concludes.

Fig 11. Electron migration diagram of the protrusion defect’s partial discharge.

Two points need to be pointed out. Firstly, the voltage on the air gap is not decreased to 0, but to an extinction voltage [24]. In addition, there are still a lot of residual charges on the void wall, and the reverse electric field formed by these residual charges also plays a role in weakening the discharge.

# 4. Discussion

## 4.1. Physical process of protrusion defect discharge

For protrusion defects, when the applied voltage is unipolar, the charge injected into the polymer at the tip of the protrusion will be trapped by the trap, and the energy released in this process will be transferred to electrons through resonance, prompting the formation of hot electrons, and then the hot electrons will collide with the polymer molecular chain, resulting in chain breakage. At the same time, the free radical generated by the molecular chain break has a certain catalytic effect on the degradation of polymer, which leads to the expansion of the degradation range of polymer with the participation of oxygen, and promotes the formation of low-density region. The increase of electron free travel in the low-density region promotes the occurrence of collision ionization. The energy released by collision ionization further leads to polymer degradation in a wider range. In addition, because the electric field distortion at the tip is the most serious, the field strength is also the largest, and it will bear a large electro-mechanical stress, further accelerating the fracture of the XLPE molecular chain, and resulting in small cracks. Partial discharge is possible in such small cracks.

There are two conditions for partial discharge: the electric field strength in a certain place inside the defect reaches the minimum discharge field strength and the initial electron appears which can stimulate the electron collapse. When the partial discharge condition is reached, the discharge begins, the electrons form electron collapse, and the migration is quickly completed. In the migration process, some electrons combine with neutral particles to form negative ions. At the same time, due to the collision ionization in the process of electron migration, a large number of positive and negative ions will drift in the opposite direction under the action of the applied electric field and finally accumulate in the interface between the gas and the solid dielectric. The surface charge that accumulates at this interface generates a reverse electric field Er, which reduces the overall electric field E in the gas cavity and ultimately results in the termination of the discharge when the field intensity is lower than the extinction field. During the discharge interval, the surface charge will gradually decay due to the conduction along the gas-solid interface and the neutralization with gas ions, and the reverse field intensity will decrease, and the electric field in the gas cavity will gradually recover. Until the conditions for partial discharge are reached again, the next discharge begins.

It is worth noting that in this process, the migration of both electrons and positive and negative ions is completed within a few ns, which is far less than the minimum discharge interval of 0.1 ms. Therefore, the actual determining factor of the discharge interval is the speed of surface charge decay and the discharge delay. The discharge delay here refers to the moment when the minimum discharge field intensity is exceeded and the time difference when partial discharge occurs.

If the external high voltage is maintained for enough time, the air gap at the tip of the protrusion defect will continue to develop under continuous partial discharge. The final result of this process is the development of electrical tree defects, which eventually lead to the breakdown of cable insulation.

## 4.2. Evolution mechanism analysis of protrusion defect discharge

As shown in the above analysis, when a small air gap is generated near the prominent defect location due to charge injection and a large degree of electric field distortion, the air gap will gradually become larger under partial discharge, and this process is agreed to accompany the development of partial discharge. Therefore, the relationship between discharge capacity development and defects is explored respectively from the numerical calculation and the three-capacitance model.

The calculation is based on the simulation of 2.4: since the whole process of charge transfer of a discharge has been numerically simulated in 2.4. And the essence of electricity is the movement of charged particles. Therefore, the pulse current of partial discharge can be calculated by calculating the power of the electric field force of the charged particles in a discharge divided by the voltage at both ends. Just according to the following formula [25]:

$$\text{I }\text{= }\frac{\text{e}}{\text{U}_{\text{a}}}\iiint(\text{n}_{\text{p}}\text{W}_{\text{p}}\text{-n}_{\text{e}}\text{W}_{\text{e}}\text{-}\text{n}_{\text{n}}\text{W}_{\text{n}})\text{*E}\text{dV} \text{(9)}$$

$$\text{W}_{\text{e,p,n}}\text{ =}\vec{\text{ E}}\text{ * }\text{μ}_{\text{e,p,n}} \text{ }\text{(}\text{10}\text{)}$$

where *I* is the discharge current, *e* is the electron charge, $\text{U}_{\text{a}}$ is the voltage at both ends of the channel in the simulation process, $\vec{\text{ E}}$ is the electric field strength, and $\text{n}_{\text{e,p,n}}$ is the concentration of charged particles. $\text{μ}_{\text{e,p,n}}$ is the mobility of charged particles.$\text{W}_{\text{e,p,n}}$ represents the work done by the electric field force on the particles.

It is worth noting that the specific values of the physical quantities presented here can be obtained by calculating the simulation model in Part 2.4.

The length of the air domain calculated in the geometric part of the Part 2.4 model is modified from 0.5 mm to 1 mm, which means that the discharge channel is extended from 0.5 mm to 1 mm. This gives the result of Fig 12: the time to obtain the maximum discharge current also increases from 1.9 ns to 4.3 ns. At the same time, the maximum discharge current increased from 12.05 nA to 19.55 nA.

Fig 12. Discharge currents for 0.5 mm and 1 mm discharge paths.

It can be seen from the simulation that the length of the discharge path greatly affects the discharge quantity, the mechanism is explored below.

The equivalent capacitance model of the discharge channel with protrusion defect in the DC field is established [26], just as in Fig 13. The capacitance at the defect of the cable is *C*_3,_ and the leakage resistance is *R*_3_*, C*_1_*, C*_2_*,* and *R*_1_*, R*_2_ are the capacitance and leakage resistance of the part of the insulation in parallel and series with the defect, respectively. *U* is the voltage at both ends of the sample.

Fig 13. Equivalent circuit for partial discharge of cable defects.

When the defect is discharged immediately, the instantaneous voltage drop of *U_g_* is generated at the defect, and the electric field is alternating electric field at this time, the leakage resistance in the circuit can be ignored, and the voltage variation is distributed on *C*_1_ and *C*_2_. Apparent discharge quantity *Q* should meet:

$$\begin{aligned} \text{ Q =}\text{ C}_{\text{2}}\text{U}_{\text{g}} \\ \text{ =}\text{C}_{\text{2}}\text{E}_{\text{b}}\text{l} \end{aligned}\text{ } \text{ }\text{(11)}$$

here, *E_b_* is the average breakdown strength in the channel during discharge; $l$ is the length of the discharge path of the defect.

Therefore, it can be concluded that the apparent discharge capacity is only determined by the channel length $l$ and the internal structure of the cable. This insulation defect will worsen in long-term continuous discharge, extending the discharge channel, and the longer discharge channel will in turn increase the discharge energy. Finally, the evolution of discharge characteristics is accelerated, and the expansion of defects is accelerated until the defects penetrate the insulation layer, making the defects completely ineffective.

## 4.3. Analysis of protrusion defect deterioration process

One step further, analysis of the development of defects based on the changes in discharge characteristics during different discharge stages. Fig 14 present the average discharge frequency and average discharge quantity of the protrusion defect at different stages. It can be observed from the averaged discharge frequency and quantity that, with the progression of discharge stages for the protrusion defect, both the averaged discharge frequency and quantity consistently increase. Specifically, the average discharge frequency rises from 82 events per minute in the first stage to 438 events per minute in the fourth stage, while the average discharge quantity increases from 41 pC in the first stage to 102 pC in the fourth stage. This changing trend aligns with the typical variation in discharge frequency and quantity observed during the DC treeing process under needle-plane electrodes. Although the protrusion of the protrusion defect in this study does not serve as a metal electrode, it may still induce electric treeing.

|  |  |
| --- | --- |
| (a) Average discharge | (b) Average discharge frequency |

Fig 14. Average discharge and frequency of the protrusion defect at different stages.

It is speculated that, under the influence of the applied electric field, the semi-conductive protrusion is likely to inject charges into the interior of the insulation, which is subsequently scattered and trapped after multiple collisions. This process is accompanied by the release of energy. The energy released from charge trapping is transferred to electrons in the conduction band in a non-radiative form, converting them into hot electrons [27]. The impact of hot electrons on polymer molecular chains leads to their rupture, forming electric tree channels [28,29]. Once an electric tree channel is formed, high-frequency localized discharges within this channel continually bombard the cross-linked polyethylene molecular chains ahead, resulting in the growth of the electric tree [30]. Longer discharge paths imply more collision ionization and electron avalanches, leading to an increase in discharge quantity and greater discharge energy. This is consistent with the sharp increase in cumulative discharge quantity and its rate of change observed in the fourth stage of discharge.

# 5. Conclusions

In this paper, the evolution of partial discharge characteristics of a crosslinked polyethylene cable with the protrusion defect under long-term applied DC voltage is studied. The partial discharge of semiconductor defects in XLPE insulation will damage the cable insulation and reduce the service life of the cable. But at the same time, we can infer the defect development of the actual cable from the evolution of discharge through the relationship between the discharge amount of partial discharge and the defect channel, to assist in judging the insulation performance of the cable: When the change of discharge amplitude is not obvious, the insulation performance of the cable changes little, when the cumulative discharge has an obvious rapid upward trend, and the discharge amplitude is also increasing rapidly, which indicates that the protrusion defect in the cable is likely to be in the fourth stage of discharge, and the cable is about to be damaged. The evolution process of partial discharge characteristics of the protrusion defect is studied in this paper, and further work can be done on other defects, such as air gap defects and fouling defects.

# Reference

1. Satapathy, S.S. and Kumar, N. (2019). Modulated perturb and observe maximum power point tracking algorithm for solar PV energy conversion system. In 2019 3rd International Conference on Recent Developments in Control, Automation & Power Engineering (RDCAPE), pp. 345-350.
2. Satapathy, S.S. and Kumar, N. (2020). Framework of maximum power point tracking for solar PV panel using WSPS technique. *IET Renewable Power Generation*, *14*(10), 1668-1676.
3. Kumar, N., Saxena, V., Singh, B. and Panigrahi, B.K. (2023). Power quality improved grid-interfaced PV assisted onboard EV charging infrastructure for smart households consumers. *IEEE Transactions on Consumer Electronics*.
4. Kumar, N., Singh, H. K., and Niwareeba, R. (2023). Adaptive control technique for portable solar powered EV charging adapter to operate in remote location. *IEEE Open Journal of Circuits and Systems*, *4*, 115-125.
5. Kumar, N., Saxena, V., Singh, B. and Panigrahi, B.K. (2020). Intuitive control technique for grid connected partially shaded solar PV‐based distributed generating system. *IET Renewable Power Generation*, *14*(4), pp.600-607.
6. Feng, A., Zhu, X., Chen, Y., Liu, P., Han, F., Zu, Y., ... and Bi, P. (2024). Functional Biomass‐derived Materials for the Development of Sustainable Batteries. *ChemElectroChem*, e202400086.
7. Kumar, N. (2023). EV charging adapter to operate with isolated pillar top solar panels in remote locations. *IEEE Transactions on Energy Conversion*.
8. Kumar, N., Mulo, T., and Verma, V. P. (2013). Application of computer and modern automation system for protection and optimum use of High voltage power transformer. In *2013 International Conference on Computer Communication and Informatics* (pp. 1-5). IEEE.
9. Wang, Z., Yang, M., Cheng, Y., Liu, J., Xiao, B., Chen, S., ... and Wu, H. (2019). Dielectric properties and thermal conductivity of epoxy composites using quantum-sized silver decorated core/shell structured alumina/polydopamine. *Composites Part A: Applied Science and Manufacturing*, *118*, 302-311.
10. Ren, J., Jiang, G., Wang, Z., Qing, Q., Teng, F., Jia, Z., ... and Jia, S. (2024). Highly thermoconductive and mechanically robust boron nitride/aramid composite dielectric films from non-covalent interfacial engineering. *Advanced Composites and Hybrid Materials*, *7*(1), 5.
11. Wang, Z., Wang, X., Wang, S., He, J., Zhang, T., Wang, J., and Wu, G. (2021). Simultaneously enhanced thermal conductivity and dielectric breakdown strength in sandwich AlN/epoxy composites. *Nanomaterials*, *11*(8), 1898.
12. Chen, S., Cheng, Y., Xie, Q., Xiao, B., Wang, Z., Liu, J., and Wu, G. (2019). Enhanced breakdown strength of aligned-sodium-titanate-nanowire/epoxy nanocomposites and their anisotropic dielectric properties. *Composites Part A: Applied Science and Manufacturing*, *120*, 84-94.
13. Pan, C., Kou, K., Jia, Q., Zhang, Y., Wu, G., and Ji, T. (2017). Improved thermal conductivity and dielectric properties of hBN/PTFE composites via surface treatment by silane coupling agent. *Composites Part B: Engineering*, *111*, 83-90.
14. P. H. F. Morshuis and J. J. Smit. (2005). Partial discharges at DC voltage: their mechanism, detection and analysis. in IEEE Transactions on Dielectrics and Electrical Insulation, vol. 12, no. 2, pp. 328-340.
15. C. Mayoux. (2000). Degradation of insulating materials under electrical stress. in IEEE Transactions on Dielectrics and Electrical Insulation. vol. 7, no. 5, pp. 590-601.
16. A. Ishibashi, T. Kawai, S. Nakagawa, H. Muto, S. Katakai, K. Hirotsu et al., (1998). A study of treeing phenomena in the development of insulation for 500 kV XLPE cables. in IEEE Transactions on Dielectrics and Electrical Insulation, vol. 5, no. 5, pp. 695-706.
17. G. Lin, H. Zhang, M. Zhou, Y. Wang and Y. Yin, (2022). Partial Discharge Characteristics of Electrical Tree in XLPE during DC and Voltage Drop. *2022 IEEE International Conference on High Voltage Engineering and Applications (ICHVE)*, Chongqing, China, 2022, pp. 1-4.
18. X. Zhu, J. Wu, Y. Wang and Y. Yin, (2020). Characteristics of partial discharge and AC electrical tree in XLPE and MgO/XLPE nanocomposites. in *IEEE Transactions on Dielectrics and Electrical Insulation*, vol. 27, no. 2, pp. 450-458.
19. H. Zhang, M. Chen, W. Li, G. Lin and Y. Yin, (2022). A Proposed Diagnostic Procedure for DC Electrical Tree Damage in XLPE. in *IEEE Transactions on Dielectrics and Electrical Insulation*, vol. 29, no. 4, pp. 1234-1241.
20. T. N. Tran, I. O. Golosnoy, P. L. Lewin, and G. E. Georghiou, (2011). Numerical modelling of negative discharges in air with experimental validation. J. Phys. D: Appl. Phys., vol. 44, no. 1, p. 015203.
21. P. Sattari, G. S. P. Castle, and K. Adamiak, (2011). Numerical Simulation of Trichel Pulses in a Negative Corona Discharge in Air. IEEE Trans. on Ind. Applicat., vol. 47, no. 4, pp. 1935–1943.
22. Woo Seok Kang, Jin Myung Park, Yongho Kim, and Sang Hee Hong, (2003). Numerical study on influences of barrier arrangements on dielectric barrier discharge characteristics. IEEE Trans. Plasma Sci., vol. 31, no. 4, pp. 504–510.
23. C. Pan, J. Tang, and K. Wu, (2016). The Effect of PD Process on the Accumulation of Surface Charges. IEEE Trans. Plasma Sci., vol. 44, no. 11, pp. 2545–2552.
24. Zhao, L., Su, J., Pan, Y., Li, R., Zheng, L., Zhang, Y., Wu, X. and Gao, P., (2017). Calculation on heating effect due to void discharge in polymers in cumulative breakdown process. IEEE Transactions on Dielectrics and Electrical Insulation,vol. 24, no. 5, pp. 3113-3121.
25. N. Sato, (1980). Discharge current induced by the motion of charged particles. J. Phys. D: Appl. Phys., vol. 13, no. 1, pp. L3–L6, Jan. 1980.
26. X. Shi, B. Qiao, Z. Zheng, G. Lin, Y. Wang and Y. Yin, (2023). Effect of Discharge Amplitude on Electrical Trees Based on Three-Capacitance Model. *2023 3rd International Conference on Energy Engineering and Power Systems (EEPS)*, Dali, China, 2023, pp. 511-514.
27. S. Peng, X. Zhu, J. Wu and Y. Yin, (2020). Characteristics of Partial Discharge in XLPE during Electrical Tree Initiation Process under Different Temperatures. 2020 International Symposium on Electrical Insulating Materials (ISEIM), Tokyo, Japan, 2020, pp. 561-564.
28. F. Guo, Y. Wang, W. Li, Z. Ma, J. Wu, Y. Yin, *et al*., (2018). Growth Characteristics of Electrical Tree in Crosslinked Polyethylene under Direct-Current Voltage. *2018 Condition Monitoring and Diagnosis (CMD)*, Perth, WA, Australia, 2018, pp. 1-4.
29. X. Zhu, Y. Wang, W. Li, Y. Zhao, J. Wu, Y. Yin *et al*., (2018). Electrical Tree Growth in Polyethylene during the DC Voltage Rise and the Influence of the Tree Defect Size. *2018 Condition Monitoring and Diagnosis (CMD)*, Perth, WA, Australia, 2018, pp. 1-4.
30. X. Zhu, J. Wu, Y. Wang, and Y. Yin, (2019). Characteristics of electrical tree defect during the growth period in high-voltage DC cable under stepped DC voltage. IET Gener. Transm. Distrib., 13: 3195-3201.
