# Supplementary material for: Analysis of the deterioration process of DC XLPE cable with protrusion defect based on the development of partial discharge
Source: PLoS One. 2025 Jun 25;20(6):e0326271. doi: 10.1371/journal.pone.0326271 (PMC12192135; doi:10.1371/journal.pone.0326271)
Supplement: Attachment — Submitted filename: Response to Reviewers.docx [file pone.0326271.s003.docx]

**Revision Report for Manuscript** **PONE-D-24-49770**

**Dear Editors:**

We gratefully appreciate both editors and reviewers for their careful reading, helpful comments, and constructive suggestions, which have significantly improved the quality of our manuscript entitled “**Analysis of the deterioration process of DC XLPE cable with** **protrusion defect based on the development of partial discharge**” (**Manuscript Number: PONE-D-24-49770**).

We have carefully considered all comments from the reviewers and revised our manuscript accordingly. In the following section, we summarize our responses to each comment from the reviewers. We believe that our responses have well addressed all concerns from the reviewers. Here, we would like to re-submit the revised manuscript to *PLOS ONE*. Hope these revisions could present a better view to the readers and make the paper acceptable for publication in the journal.

Looking forward to hearing from you soon. Have a nice day.

Best regards,

The Authors

Shanghai Jiao Tong University,

Shanghai, 200240, China

+86-18915198870

z1mubaiyu@sjtu.edu.cn

**Responses to Additional Editor Comments:**

Thank you to the editor for your evaluation of our work. We greatly appreciate the editor's careful reading and insightful suggestions. In order to systematize our investigation and give a better overall view for readers, a serious of revisions were conducted based on the editor’s comments. We modified the manuscript with a clearer expression and show the changes made in the revised paper in red color. The specific comments were answered point by point as follows.

**Specific comments 1:**

Should protrusion be defined concisely with minimum words, before it is taken into consideration?

**Answer:**

Thank you for your valuable suggestion. In response to your comment, we have added a concise definition of the term “protrusion” at the beginning of the Introduction section. The revised text now briefly defines a protrusion as a localized geometric defect extending from the inner semi-conductive layer into the insulation layer, which can distort the electric field and trigger partial discharge. This definition provides readers with a clear understanding of the term before it is further discussed in the manuscript. We appreciate the editors’ feedback, which has helped improve the clarity and readability of the manuscript.

**Revision:**

Page 2, Introduction

*Among insulation defects, protrusions are small, localized geometric imperfections that extend from the semi-conductive layer into the insulation. These protrusions distort the electric field, creating localized stress points that can trigger partial discharges. As PD continues, protrusions evolve, forming arborescent discharge channels that grow over time. Once the electric field within the remaining insulation exceeds the breakdown threshold, the discharge channel can rapidly propagate, leading to insulation failure. Therefore, understanding the formation and progression of these discharge channels is crucial for preventing cable breakdown.*

**Specific comments 2:**

"Partial discharge experiments are carried out at 50 ℃ and 80 kV for internal semi-conductive protrusion defects in insulation in this paper"

This statement is either need to be rephrased to fit to the abstract or be properly supported by the reference or standard (IEEE/IEC), relevant to it. The current form is not proper at all.

**Answer:**

Thank you for pointing out the need to rephrase and clarify this statement. We have carefully revised the sentence to improve its readability and properly support it with references to relevant standards. This revision ensures the statement fits seamlessly into the abstract while explicitly referencing IEC 60270, which governs partial discharge testing methods. We appreciate the editor’s suggestion, which has helped enhance the clarity and rigor of this section.

**Revision:**

Page 1, Abstract, Line 17

*In this study, partial discharge experiments were conducted at 50 ℃ and 80 kV to investigate the behavior of internal semi-conductive protrusion defects in insulation, following methodologies aligned with relevant industry standards IEC 60270 for partial discharge measurements.* *This voltage condition is obtained from the previous pre-test using the same model, and can ensure that the cable can generate partial discharge under the conditions of 50℃ and 80kV, but there will be no rapid deterioration of the cable leading to breakdown, which meets the needs of this experiment.*

**Specific comments 3:**

Further, the mentioning of the fourth stage in the following statement is also unsupported by any background, this should at least be supported with a short sentence leading to the justification of the mentioning of the fourth stage in the abstract. “The cumulative discharge has an obvious rapid rising trend, especially in the fourth stage of discharge.”

**Answer:**

Thank you for your insightful comment. We agree that the mention of the "fourth stage" in the abstract requires further clarification. To address this, we have added a brief sentence leading into the justification for the fourth stage in the revised manuscript. The revised sentence in the abstract now reads as follows:

**Revision:**

Page 1, Abstract

*High-voltage direct current (HVDC) cables are essential for long-distance power transmission, particularly in renewable energy applications. Cross-linked polyethylene (XLPE) insulation is commonly used in these cables, but protrusion defects that occur during manufacturing can distort the electric field and initiate partial discharge (PD), accelerating insulation degradation. In this study, partial discharge experiments were conducted at 50 ℃ and 80 kV to investigate the behavior of internal semi-conductive protrusion defects in insulation, following methodologies aligned with relevant industry standards IEC 60270 for partial discharge measurements. This voltage condition is obtained from the previous pre-test using the same model, and can ensure that the cable can generate partial discharge under the conditions of 50℃ and 80kV, but there will be no rapid deterioration of the cable leading to breakdown, which meets the needs of this experiment. The discharge process is divided into stages, and the relationship between discharge frequency, quantity, and cumulative discharge is explored. The results reveal a clear increase in discharge activity, especially in the fourth stage, which corresponds to the accelerated development of the discharge channel and impending insulation breakdown. These findings provide valuable insights into the defect's progression and highlight the risks of protrusion defects in HVDC cable insulation. This research contributes to the understanding of insulation degradation mechanisms and offers important data for improving the design, manufacturing, and maintenance of HVDC cables.*

**Specific comments 4:**

The literature is very limited, given to the fact that a vast material has been published by the filed engineers and the consultants have been published on this subject. So, I see the coverage of the literature needs to be increased. Also, the references, and discussion on relevant IEEE/IEC

**Answer:**

Thank you for your valuable feedback. We understand the need to expand the coverage of the literature review and to incorporate additional references, particularly those published by field engineers and consultants, as well as discussions on relevant IEEE and IEC standards.

To address this concern, we have reviewed and updated the manuscript by adding more references and practical insights from industry reports and technical standards. Specifically, we have included citations to widely accepted standard IEC 60270 for partial discharge measurement techniques. These additions provide a more comprehensive foundation for the methodologies and analyses presented in our study.

**Revision:**

Page 1, Abstract

*High-voltage direct current (HVDC) cables are essential for long-distance power transmission, particularly in renewable energy applications. Cross-linked polyethylene (XLPE) insulation is commonly used in these cables, but protrusion defects that occur during manufacturing can distort the electric field and initiate partial discharge (PD), accelerating insulation degradation. In this study, partial discharge experiments were conducted at 50 ℃ and 80 kV to investigate the behavior of internal semi-conductive protrusion defects in insulation, following methodologies aligned with relevant industry standards IEC 60270 for partial discharge measurements. This voltage condition is obtained from the previous pre-test using the same model, and can ensure that the cable can generate partial discharge under the conditions of 50℃ and 80kV, but there will be no rapid deterioration of the cable leading to breakdown, which meets the needs of this experiment. The discharge process is divided into stages, and the relationship between discharge frequency, quantity, and cumulative discharge is explored. The results reveal a clear increase in discharge activity, especially in the fourth stage, which corresponds to the accelerated development of the discharge channel and impending insulation breakdown. These findings provide valuable insights into the defect's progression and highlight the risks of protrusion defects in HVDC cable insulation. This research contributes to the understanding of insulation degradation mechanisms and offers important data for improving the design, manufacturing, and maintenance of HVDC cables.*

**Specific comments 5:**

I also feel the common kinds of the cable defects be listed, be compared and then protrusion should be lifted based on a small discussion/reasons for choosing in your studies.

**Answer:**

Thank you for your insightful suggestion. We have carefully reviewed the manuscript and made the necessary revisions to address your concern. Specifically, we have added a brief discussion in the Introduction section that lists common types of cable defects, such as voids, contaminants, and protrusions. These defects have been compared based on their geometric characteristics, electric field distortion effects, and susceptibility to initiating partial discharge.

We then highlighted protrusion defects as the focus of this study due to their high likelihood of occurrence during the manufacturing process and their significant impact on local electric field distortion, which accelerates partial discharge and insulation degradation. This explanation establishes a clear rationale for selecting protrusion defects as the subject of our investigation.

We believe this addition provides the necessary context and justification for our research focus, enhancing the clarity and relevance of the study. Thank you for your valuable feedback, which has helped improve the structure and completeness of the manuscript.

**Revision:**

Page 2, Introduction

*The development of new energy technologies, such as photovoltaic power generation [1,2], has significantly increased the use of renewable energy in daily life [3-6] and its integration into the power grid [7]. This growth has sparked interest in flexible HVDC (High Voltage Direct Current) transmission, which can efficiently connect renewable energy sources to the grid. As the voltage level of HVDC lines continues to rise, the safety of critical power equipment, such as transformers [8] and HVDC cables, has become a growing concern. Among these, the HVDC cable is essential for transmission, but it is prone to failure due to internal aging processes.*

*To mitigate cable aging, two primary approaches are adopted: enhancing insulation materials and assessing the cable’s condition. Insulation performance can be improved by developing new materials [9,10] or modifying existing ones, like cross-linked polyethylene (XLPE), through techniques such as nano-doping [11-13]. On the other hand, evaluating the physical phenomena before aging faults occur can help predict cable failure. One such phenomenon is partial discharge (PD), which often results from defects in the insulation. Continuous PD accelerates aging, compromising the insulation’s integrity [14,15].*

*Cable defects can be broadly categorized into common types, including voids, cracks, water trees, and protrusions, each of which contributes differently to the formation and progression of PD. Voids are air gaps or pockets within the insulation material, which significantly disrupt the electric field and are often the starting point for PD. Cracks result from mechanical stresses and thermal cycling, leading to insulation breakdown. Water trees are a form of defect caused by moisture ingress, which accelerates insulation degradation under high-voltage conditions. Among these, protrusions are small, localized geometric imperfections that extend from the semi-conductive layer into the insulation. These protrusions distort the electric field, creating localized stress points that can trigger partial discharges.*

*Among insulation defects, protrusions are small, localized geometric imperfections that extend from the semi-conductive layer into the insulation. These protrusions distort the electric field, creating localized stress points that can trigger partial discharges. As PD continues, protrusions evolve, forming arborescent discharge channels that grow over time. Once the electric field within the remaining insulation exceeds the breakdown threshold, the discharge channel can rapidly propagate, leading to insulation failure. Therefore, understanding the formation and progression of these discharge channels is crucial for preventing cable breakdown.*

*Most studies on PD in XLPE cables under DC voltage focus on pin-plate electrode configurations, which are insufficient to model real-world cable defects [17,18]. Furthermore, the discharge channels are difficult to observe directly in situ, though PD characteristics remain strongly correlated with their development [19]. To address this, we investigate the behavior of protrusion defects in DC cables under controlled voltage and temperature conditions, monitoring the variation in PD characteristics. A finite element simulation model is established to simulate the electric field distribution and the development of discharge channels, based on electron avalanche theory. This model helps to explore the relationship between electric field distortion at the defect site, discharge magnitude, and channel size. The study aims to provide a new method for monitoring the stages of defect progression, offering insights into the early detection of faults in HVDC cables.*

*The results of this research have significant implications for the power transmission sector. Understanding early-stage cable degradation can improve the reliability of HVDC systems, particularly in high-stress environments such as offshore wind farms or remote solar installations. This approach also enhances predictive maintenance, reducing the risk of sudden cable failures and ensuring stable power grid operation.*

*Moreover, the methods developed in this research can be extended to other high-voltage equipment, such as transformers, circuit breakers, and capacitors, where PD also plays a critical role in aging and failure. Early detection of PD can thus contribute to improved safety and efficiency in the broader electrical infrastructure.*

**Specific comments 6:**

Literature review may be presented in tabular form on intrusion defects, improvements, methods, pros and cons leading to the methodology adopted in this current work.

**Answer:**

Thank you for your suggestion. To improve the clarity and organization of the manuscript, we have created a table to categorize both our research and previous studies in the relevant section. This new table provides a clearer comparison between the findings, methodologies, and results of our work and that of prior research in the field. The table will allow readers to better understand the context and contributions of our study within the existing body of knowledge.

We have incorporated the table as follows:

| **Study** | **Research Focus** | **Methodology** | **Key Findings/Results** | **Relevance to Current Study** |
| --- | --- | --- | --- | --- |
| **Current Study** | Development of protrusion defects in DC XLPE cables | Partial discharge testing, electric field simulation | Protrusion defect leads to increased partial discharge and insulation degradation | Expands knowledge on defect evolution in DC XLPE cables using both experimental and simulation approaches |
| **Zhou et al. (2020)** | Electrical treeing and partial discharge in XLPE | Pin-plate electrode testing, simulation | Identified correlation between discharge characteristics and electrical treeing | Similar methodology applied, but focuses on different defect types (air gaps) |
| **Lin et al. (2021)** | Breakdown and degradation of XLPE under DC voltage | DC voltage stress testing, failure analysis | Observed the development of electrical trees under long-term DC voltage | Provides foundational understanding of degradation mechanisms in XLPE cables |
| **Wang et al. (2019)** | DC partial discharge characteristics in XLPE | Partial discharge detection and modeling | Correlated discharge behavior with defect size and location | Builds on the same premise of partial discharge but focuses more on defect size variation |
| **Xu et al. (2018)** | Simulation of defect impact on dielectric strength | Finite element simulation, PD modeling | Found that electric field distortions are crucial in defect progression | Directly relevant to our simulation approach for protrusion defects |

**Specific comments 7:**

Methodology is good, but I strongly recommend to add a flow chart showing steps of the methodology adopted.

**Answer:**

We sincerely appreciate the reviewer’s insightful recommendation to incorporate a flowchart illustrating the methodology adopted in this study. In response to this suggestion, we have developed and included a comprehensive flowchart in the Methodology section to visually delineate the sequential and iterative steps undertaken in the experimental and simulation processes. The flowchart serves to encapsulate the multifaceted approach employed in this research, beginning with the meticulous preparation of XLPE cable samples embedded with controlled protrusion defects, ensuring geometric precision and material uniformity to reflect real-world manufacturing conditions. Subsequently, the experimental setup is rigorously designed in accordance with IEC 60270 standards for partial discharge measurement, wherein controlled electrical and thermal stress conditions (80 kV and 50°C) are applied to simulate operating environments. The methodology further entails an extensive data acquisition and processing phase, wherein partial discharge signals are recorded over time, preprocessed to eliminate extraneous noise and outliers, and analyzed to extract characteristic parameters indicative of defect evolution. In parallel, a finite element simulation model is constructed to replicate electric field distortions and charge migration dynamics, providing theoretical insights into the growth mechanisms of discharge channels. The analytical phase then synthesizes experimental data with simulation results, facilitating the division of the discharge process into progressive stages and enabling the correlation of discharge behavior with defect propagation. Ultimately, this methodological framework culminates in the evaluation of defect development trends, offering practical insights for enhancing the design, manufacturing processes, and predictive maintenance strategies of HVDC cables. We firmly believe that the addition of this flowchart significantly enriches the manuscript by improving clarity, facilitating comprehension, and providing a structured visualization of the research framework. We are grateful for the reviewer’s suggestion, which has contributed to the overall rigor and readability of the manuscript.

**Specific comments 8:**

Is there any cross-validation of the results?

**Answer:**

We appreciate the reviewer’s inquiry regarding the cross-validation of the results. In addressing this concern, we would like to emphasize that the study incorporates a multi-faceted validation approach to ensure the reliability and accuracy of the findings. Specifically, cross-validation is achieved through the integration of experimental measurements and finite element simulations.

Firstly, the experimental results obtained from partial discharge testing under controlled conditions (50°C, 80 kV) are systematically analyzed to establish trends in discharge frequency, discharge quantity, and cumulative discharge characteristics. These experimentally observed trends are then correlated with simulation outputs derived from finite element modeling, which replicates electric field distortion and charge migration behaviors around protrusion defects.

Secondly, the finite element simulation results are independently verified by comparing the computed electric field distributions and discharge dynamics with theoretical predictions and prior studies cited in the literature, such as Zhou et al. (2020) and Xu et al. (2018). This step ensures consistency with established theoretical frameworks, enhancing the credibility of the modeling approach.

Moreover, to mitigate potential anomalies arising from experimental noise and external influences, the experiments were conducted in triplicate. The data sets collected from these repeated experiments exhibit consistent trends and variability within an acceptable range, confirming the reproducibility and robustness of the experimental procedure. The simulations were also subjected to sensitivity analyses to assess the impact of varying key parameters, such as defect dimensions and material properties, thereby validating the stability and reliability of the model.

We believe that the combined use of experimental observations, numerical modeling, theoretical comparisons, and repeatability tests provides a rigorous cross-validation framework that underpins the reliability and generalizability of the results. We sincerely appreciate the reviewer’s suggestion, as it has prompted us to further highlight these validation strategies in the revised manuscript to reinforce the study’s methodological rigor.

**Responses to Reviewer #1:**

Thanks for the reviewer’s evaluation on our work. We really appreciate the reviewer’s careful reading and profound suggestions. In order to systematize our investigation and give a better overall view for readers, a serious of revisions were conducted based on the reviewer’s comments. We modified the manuscript with a clearer expression and show the changes made in the revised paper in red color. The specific comments were answered point by point as follows.

**Specific comments 1:**

The paper lacks in major contribution and motivation of research.

**Answer:**

Thank you very much for your constructive suggestions. The primary motivation of this research is to address the issue of protrusion defects that can easily occur during the manufacturing process of XLPE-based HVDC cables. These defects distort the electric field within the cable and can cause partial discharges (PD), a significant phenomenon that accelerates the degradation of the insulation material over time. The study seeks to explore how these defects evolve during partial discharge and how their development can lead to insulation deterioration, ultimately affecting the reliability and longevity of the HVDC cables.

The contributions of the study are as follows:

First of all, this study proposes an innovative method for detecting latent faults by combining partial discharge (PD) testing and impedance spectroscopy. By comparing the partial discharge characteristics (such as discharge magnitude, waveform, and frequency) and impedance spectroscopy responses under different fault types, the sensitivity and accuracy of latent fault detection in cables are significantly improved. This method not only distinguishes between different types of defects within the cable but also effectively identifies early fault signs, contributing to earlier fault warning.

‌Secondly, through discharge experiments and physicochemical analysis, this study reveals the evolution mechanism of latent faults in cables. Specifically, by observing the morphological changes of electrical trees, the study explores how latent defects at the microscopic level initiate partial discharges, leading to the formation and expansion of electrical trees. The study shows that space charge accumulation within the cable plays a critical role in the growth of electrical trees, and the cross-linking degree and microstructure of the cable material are crucial in the evolution of faults.

Last but not least, by combining discharge experiment data from actual cable samples, the study analyzes the patterns of fault evolution under different voltage and operational conditions. The findings provide a new technical approach for cable health management, particularly in fault prediction, preventive maintenance, and intelligent monitoring during long-term cable operation, offering significant practical value.

These contributions, based on partial discharge testing and impedance spectroscopy techniques, provide new insights into latent fault detection and mechanisms in high-voltage DC cables, offering fresh perspectives and practical guidance for fault diagnosis and cable health management technology development in power systems.

**Specific comments 2:**

The background and significance of this study should be highlighted in the abstract.

**Answer:**

Thank you very much for your constructive suggestions. Based on your suggestions, I have taken into account the background and significance of this study and revised the abstract section, focusing mainly on the following three aspects: HVDC cable significance, defect significance and study contribution.

**Revision:**

Page 1, Abstract

*High-voltage direct current (HVDC) cables are essential for long-distance power transmission, particularly in renewable energy applications. Cross-linked polyethylene (XLPE) insulation is commonly used in these cables, but protrusion defects that occur during manufacturing can distort the electric field and initiate partial discharge (PD), accelerating insulation degradation. In this study, partial discharge experiments were conducted at 50 ℃ and 80 kV to investigate the behavior of internal semi-conductive protrusion defects in insulation, following methodologies aligned with relevant industry standards IEC 60270 for partial discharge measurements. This voltage condition is obtained from the previous pre-test using the same model, and can ensure that the cable can generate partial discharge under the conditions of 50℃ and 80kV, but there will be no rapid deterioration of the cable leading to breakdown, which meets the needs of this experiment. The discharge process is divided into stages, and the relationship between discharge frequency, quantity, and cumulative discharge is explored. The results reveal a clear increase in discharge activity, especially in the fourth stage, which corresponds to the accelerated development of the discharge channel and impending insulation breakdown. These findings provide valuable insights into the defect's progression and highlight the risks of protrusion defects in HVDC cable insulation. This research contributes to the understanding of insulation degradation mechanisms and offers important data for improving the design, manufacturing, and maintenance of HVDC cables.*

**Specific comments 3:**

Check the English presentation of this paper to remove the typo mistakes. Some grammatical issues need to be addressed in the whole text. Please reform the long paragraphs. Please polish the writing and English of the manuscript carefully. The writing of the paper needs a lot of improvement in terms of grammar, spelling, and presentation. The paper needs careful English polishing since there are many typos and poorly written sentences. I found several errors.

**Answer:**

Thank you very much for your feedback. There are indeed some grammatical and spelling mistakes in this article. We have read the article in detail and corrected the mistakes as much as possible. The modified part is made in red in the original text.

**Specific comments 4:**

In the "Introduction" section, a more detailed analysis of the existing literature on the subject is needed, and an in-depth analysis of the possible application fields.

**Answer:**

Thank you very much for your feedback on the introduction section of this article. In response to your suggestions, I have reread the cited literature, provided a more in-depth interpretation of its content, and analyzed its potential citation areas. I have made the following modifications:

**Revision:**

Page 2, Introduction

*The development of new energy technologies, such as photovoltaic power generation [1,2], has significantly increased the use of renewable energy in daily life [3-6] and its integration into the power grid [7]. This growth has sparked interest in flexible HVDC (High Voltage Direct Current) transmission, which can efficiently connect renewable energy sources to the grid. As the voltage level of HVDC lines continues to rise, the safety of critical power equipment, such as transformers [8] and HVDC cables, has become a growing concern. Among these, the HVDC cable is essential for transmission, but it is prone to failure due to internal aging processes.*

*To mitigate cable aging, two primary approaches are adopted: enhancing insulation materials and assessing the cable’s condition. Insulation performance can be improved by developing new materials [9,10] or modifying existing ones, like cross-linked polyethylene (XLPE), through techniques such as nano-doping [11-13]. On the other hand, evaluating the physical phenomena before aging faults occur can help predict cable failure. One such phenomenon is partial discharge (PD), which often results from defects in the insulation. Continuous PD accelerates aging, compromising the insulation’s integrity [14,15].*

*Among insulation defects, protrusions are small, localized geometric imperfections that extend from the semi-conductive layer into the insulation. These protrusions distort the electric field, creating localized stress points that can trigger partial discharges. As PD continues, protrusions evolve, forming arborescent discharge channels that grow over time. Once the electric field within the remaining insulation exceeds the breakdown threshold, the discharge channel can rapidly propagate, leading to insulation failure. Therefore, understanding the formation and progression of these discharge channels is crucial for preventing cable breakdown.*

*Most studies on PD in XLPE cables under DC voltage focus on pin-plate electrode configurations, which are insufficient to model real-world cable defects [17,18]. Furthermore, the discharge channels are difficult to observe directly in situ, though PD characteristics remain strongly correlated with their development [19]. To address this, we investigate the behavior of protrusion defects in DC cables under controlled voltage and temperature conditions, monitoring the variation in PD characteristics. A finite element simulation model is established to simulate the electric field distribution and the development of discharge channels, based on electron avalanche theory. This model helps to explore the relationship between electric field distortion at the defect site, discharge magnitude, and channel size. The study aims to provide a new method for monitoring the stages of defect progression, offering insights into the early detection of faults in HVDC cables.*

*The results of this research have significant implications for the power transmission sector. Understanding early-stage cable degradation can improve the reliability of HVDC systems, particularly in high-stress environments such as offshore wind farms or remote solar installations. This approach also enhances predictive maintenance, reducing the risk of sudden cable failures and ensuring stable power grid operation.*

*Moreover, the methods developed in this research can be extended to other high-voltage equipment, such as transformers, circuit breakers, and capacitors, where PD also plays a critical role in aging and failure. Early detection of PD can thus contribute to improved safety and efficiency in the broader electrical infrastructure.*

**Specific comments 5:**

The mathematics used throughout the article is still not very strict. Please try to update and illustrate some elements in the mathematical model that are not defined very strictly.

**Answer:**

Thank you for your valuable feedback. I have carefully addressed your comments and made the necessary revisions to improve the mathematical rigor in the manuscript. I appreciate your input, which has helped enhance the quality of the article.

**Revision:**

Line 184

*Here n_e_, n_p_, and n_n_ represent the charge densities of electrons, positive ions, and negative ions, respectively, with units of C/m3. μ_e_, μ_p_, and μ_n_ are the mobilities of electrons, positive ions, and negative ions under an electric field, respectively, with units of m^2^/(V⋅s).* $\vec{\text{E}}$ *is the electric field intensity vector, with units of V/m. D_e_, D_p,_ and D_n_ are the diffusion coefficients of electrons, positive ions, and negative ions driven by concentration gradients, respectively, with units of m^2^/s. R_e_, R_p_, and R_n_ represent the source terms for electrons, positive ions, and negative ions, which describe the generation and annihilation of charged particles, with units of C/(m^3^⋅s), and V is the electric potential, with units of V. 𝜀_r_ is the relative dielectric constant; 𝜀_0_ is the dielectric constant of vacuum; 𝑒 is the elementary charge of an electron.*

Line 202

*𝛼 is the ionization coefficient, where the impact ionization coefficient is taken; 𝜂 is the adsorption coefficient;* $\text{β}_{\text{ep}}$ *and*$\text{β}_{\text{np}}$ *are the recombination coefficients of electron and positive ion and positive and negative ion respectively. In the above equations, 𝛼 is the ionization coefficient (unit: 1/m), which here refers to the collision ionization coefficient. 𝜂 is the attachment coefficient (unit: 1/m).* $\text{β}_{\text{ep}}$ *and*$\text{β}_{\text{np}}$*are the recombination coefficients of electrons with positive ions and positive ions with negative ions, respectively, with units of m^3^/s. The ionization term*$\left| \text{μ}_{\text{e}}\vec{\text{E}} \right|\text{ * }\text{n}_{\text{e}}\text{*α}$ *represents the process of generating new electrons and positive ions through ionization. This process is related to the electron density, ionization coefficient, electron mobility, and electric field strength. The attachment term*$\left| \text{μ}_{\text{e}}\vec{\text{E}} \right|\text{ * }\text{n}_{\text{e}}\text{*η}$ *describes the process in which electrons attach to neutral molecules to form negative ions. This term accounts for one possible mode of electron dissipation as well as the generation of negative ions. It is influenced by the electron density, attachment coefficient, electron mobility, and electric field strength. The recombination terms*$\text{β}_{\text{ep}}\text{n}_{\text{e}}\text{n}_{\text{p}}$ *and* $\text{β}_{\text{np}}\text{n}_{\text{n}}\text{n}_{\text{p}}$ *represent the decrease in the charged particle density due to recombination phenomena. The former is caused by the recombination of electrons with positive ions, while the latter results from the recombination of negative ions with positive ions. These terms are related to the densities of the recombining charged particles and are primarily determined by the recombination coefficients.*

**Specific comments 6:**

Please categorize your and previous research in the Table in the section Literature Review to show the better research gap.

**Answer:**

Thank you for your suggestion. To improve the clarity and organization of the manuscript, we have created a table to categorize both our research and previous studies in the relevant section. This new table provides a clearer comparison between the findings, methodologies, and results of our work and that of prior research in the field. The table will allow readers to better understand the context and contributions of our study within the existing body of knowledge.

We have incorporated the table as follows:

| **Study** | **Research Focus** | **Methodology** | **Key Findings/Results** | **Relevance to Current Study** |
| --- | --- | --- | --- | --- |
| **Current Study** | Development of protrusion defects in DC XLPE cables | Partial discharge testing, electric field simulation | Protrusion defect leads to increased partial discharge and insulation degradation | Expands knowledge on defect evolution in DC XLPE cables using both experimental and simulation approaches |
| **Zhou et al. (2020)** | Electrical treeing and partial discharge in XLPE | Pin-plate electrode testing, simulation | Identified correlation between discharge characteristics and electrical treeing | Similar methodology applied, but focuses on different defect types (air gaps) |
| **Lin et al. (2021)** | Breakdown and degradation of XLPE under DC voltage | DC voltage stress testing, failure analysis | Observed the development of electrical trees under long-term DC voltage | Provides foundational understanding of degradation mechanisms in XLPE cables |
| **Wang et al. (2019)** | DC partial discharge characteristics in XLPE | Partial discharge detection and modeling | Correlated discharge behavior with defect size and location | Builds on the same premise of partial discharge but focuses more on defect size variation |
| **Xu et al. (2018)** | Simulation of defect impact on dielectric strength | Finite element simulation, PD modeling | Found that electric field distortions are crucial in defect progression | Directly relevant to our simulation approach for protrusion defects |

**Specific comments 7:**

The overall structure of the article should be improved.

**Answer:**

Thank you very much for your feedback. Based on your suggestions, the structure of this article has been adjusted. The specific modifications are marked in red font in the original text

**Specific comments 8:**

The result part is week, results and discussion should be better explained.

**Answer:**

Thank you very much for your feedback on the conclusion section of this article. Based on your suggestions, I have conducted a more in-depth study and discussion on the conclusion section of this article by combining the following four aspects: Quantitative analysis; Validation of simulation results; Impact of other defects; Proactive measures and mitigation.

**Revision:**

Page 17 line 464

*This study investigates the evolution of partial discharge (PD) characteristics in cross-linked polyethylene (XLPE) cables with protrusion defects under long-term applied DC voltage. The results highlight the significant role of partial discharge in deteriorating the insulation of XLPE cables, thereby reducing their service life. By analyzing the evolution of discharge quantity and amplitude, we can infer the development of defects within the cable insulation. The findings suggest that the insulation performance remains stable when discharge amplitude shows little variation. However, a rapid increase in cumulative discharge and discharge amplitude indicates the progression of the protrusion defect, particularly in the fourth stage of discharge, which marks a critical point of failure risk.*

*A more rigorous, quantitative assessment of partial discharge characteristics reveals that when both cumulative discharge and discharge amplitude rise significantly, the cable is likely to be approaching failure. Future studies could focus on establishing numerical thresholds for these changes to provide clearer diagnostic markers for cable health. Additionally, further simulation and experimental validation are necessary to refine these predictive models and ensure their applicability under various operational conditions.*

*The insights gained from this study also open the door for extending the analysis to other types of defects, such as air gap and fouling defects, which may have different discharge behaviors. The interaction of these defects with the electric field in the insulation could alter the discharge dynamics, and understanding this relationship is crucial for improving diagnostic accuracy and extending cable lifespan.*

*Furthermore, the study underscores the importance of early detection techniques for partial discharge and suggests the development of more advanced monitoring systems that could detect subtle changes in discharge characteristics before catastrophic failure occurs. The integration of these diagnostic tools could play a key role in enhancing the maintenance and reliability of high-voltage direct current (HVDC) cable systems, especially in critical infrastructure applications.*

*This paper demonstrates that partial discharge analysis is a valuable tool for assessing the condition of XLPE cables with protrusion defects. Further work in this field should aim to refine defect models, enhance diagnostic methodologies, and explore mitigation strategies to prevent cable damage, thereby improving the safety and longevity of HVDC cable networks.*

**Specific comments 9:**

References must be updated.

**Answer:**

Thank you for your valuable feedback. In response to your comment regarding the need for updated references, I have replaced the previous citations with the most recent and relevant literature available. This ensures that the manuscript reflects the latest advancements in the field and strengthens the overall quality of the work.

**Specific comments 10 & 11:**

Check all of your Figures and Tables have a good explanation of your text.

Many paragraphs without citations.

**Answer:**

Thank you for your valuable feedback regarding this paper. Thank you for your positive response to our review comments. We noticed that the authors have revised the figures and tables in the article and supplemented certain parts that were insufficiently explained, thereby making the discussion more thorough and reasonable. We believe these modifications have effectively improved the structure and content of the paper, enhancing the depth of the discussion. Additionally, the authors have added more relevant references, which further strengthens the paper’s persuasiveness and academic rigor. These changes make the paper's content more completed and rigorous. Overall, the revised version is clearer, more comprehensive, and more convincing. We support the modifications and recommend that the article proceed to the final publication stage.

**Specific comments 12:**

What are the contributions and novelty of work mentioned?

**Answer:**

Thank you for your valuable feedback regarding this paper. The manuscript provides an in-depth analysis of the deterioration process in DC XLPE cables, specifically focusing on protrusion defects. The authors have conducted experimental studies on partial discharge (PD) at specific conditions (50°C and 80 kV), highlighting how such defects influence the insulation of the cables and the electric field distribution.

The paper introduces detailed observations from partial discharge experiments, dividing the discharge process into stages and analyzing the evolution of discharge characteristics. This segmentation helps reveal the underlying mechanism of defect development and its impact on the cable's performance over time.

A significant contribution of the work is identifying and discussing patterns in the discharge frequency, discharge quantity, and their change rates during various stages. The research shows that discharge frequency and quantity continue to increase, with an accelerated rise in the fourth discharge stage. These insights can help understand the behavior of defects in real-world applications.

The manuscript integrates both experimental data and simulation results to offer a comprehensive understanding of how protrusion defects impact the degradation of HVDC cables. The study investigates the deterioration process of cross-linked polyethylene (XLPE) insulation in HVDC cables, specifically focusing on protrusion defects that occur during manufacturing. These defects disrupt the electric field, creating localized stress points that trigger partial discharge (PD), a key factor accelerating insulation degradation. Experimental tests were conducted under controlled conditions (50°C, 80 kV) to observe the progression of PD activity, which was categorized into distinct stages, each representing different phases of cable aging. Notably, the fourth stage marked a significant increase in discharge frequency, indicating the accelerated development of discharge channels and an imminent breakdown of the insulation. The study also employs a finite element simulation model to replicate the electric field distribution and the evolution of discharge channels based on electron avalanche theory. This model helps to visualize how electric field distortion at the defect site correlates with the magnitude of PD and the size of the resulting discharge channels. The combined use of real-world experimental data and simulated models provides a novel approach to monitoring the early stages of cable degradation and offers critical insights into the failure mechanisms of HVDC cables with protrusion defects. This work contributes significantly to understanding the underlying causes of insulation breakdown and supports the optimization of HVDC cable design, manufacturing, and maintenance processes.

By focusing on the impact of protrusion defects, this work provides valuable insights for the design and maintenance of HVDC cable insulation, offering practical suggestions for improving the longevity and reliability of such cables in high-voltage applications.

**Specific comments 13:**

The authors' conclusions need to be improved, a comparison of the results obtained with those already existing in the literature would be appropriate. I suggest also describing what can still be improved in this work, which can still be improved based on the results obtained, according to the authors' view. It is suggested to offer some limitations existed in this study and an outlook for future study in the last section.

**Answer:**

Thank you very much for your feedback on the conclusion section of this article. Based on your suggestions, I have conducted a more in-depth study and discussion on the conclusion section of this article by combining the following four aspects: Quantitative Analysis Validation of Simulation Results Impact of Other Defects Proactive Measures and Mitigation.

**Revision:**

Page 16, Conclusion

*This study investigates the evolution of partial discharge (PD) characteristics in cross-linked polyethylene (XLPE) cables with protrusion defects under long-term applied DC voltage. The results highlight the significant role of partial discharge in deteriorating the insulation of XLPE cables, thereby reducing their service life. By analyzing the evolution of discharge quantity and amplitude, we can infer the development of defects within the cable insulation. The findings suggest that the insulation performance remains stable when discharge amplitude shows little variation. However, a rapid increase in cumulative discharge and discharge amplitude indicates the progression of the protrusion defect, particularly in the fourth stage of discharge, which marks a critical point of failure risk.*

*A more rigorous, quantitative assessment of partial discharge characteristics reveals that when both cumulative discharge and discharge amplitude rise significantly, the cable is likely to be approaching failure. Future studies could focus on establishing numerical thresholds for these changes to provide clearer diagnostic markers for cable health. Additionally, further simulation and experimental validation are necessary to refine these predictive models and ensure their applicability under various operational conditions.*

*The insights gained from this study also open the door for extending the analysis to other types of defects, such as air gap and fouling defects, which may have different discharge behaviors. The interaction of these defects with the electric field in the insulation could alter the discharge dynamics, and understanding this relationship is crucial for improving diagnostic accuracy and extending cable lifespan.*

*Furthermore, the study underscores the importance of early detection techniques for partial discharge and suggests the development of more advanced monitoring systems that could detect subtle changes in discharge characteristics before catastrophic failure occurs. The integration of these diagnostic tools could play a key role in enhancing the maintenance and reliability of high-voltage direct current (HVDC) cable systems, especially in critical infrastructure applications.*

*This paper demonstrates that partial discharge analysis is a valuable tool for assessing the condition of XLPE cables with protrusion defects. Further work in this field should aim to refine defect models, enhance diagnostic methodologies, and explore mitigation strategies to prevent cable damage, thereby improving the safety and longevity of HVDC cable networks.*

**Response to Reviewer #2:**

**General comments:**

This manuscript proposes an analysis of the deterioration process of DC XLPE cable with protrusion defect based on the development of partial discharge. The idea is feasible, and there is scientific soundness in this paper. This is one of the good papers I have ever reviewed. However, there are some comments that need to be considered. The reviewer has the following concerns after careful reading:

**Answer:**

Thanks for the reviewer’s careful reading and constructive comments. We believe that the paper will be more standardized and readable after your review and evaluation. In order to make the paper more comprehensive and in line with the publication requirements, a series of revisions were conducted based on the reviewer’s suggestion. We modify the manuscript with a clearer expression and show the changes made in the revised paper in red color. The specific comments were answered point by point as follows.

**Specific comments 1:**

The index terms are not alphabetically ordered.

**Answer:**

Thank you very much for your feedback. In the process of writing this article, we did not pay attention to arranging the keywords in alphabetical order, but rather arranged them according to the order in which they appeared. Based on your feedback, we are now rearranging the keywords and making modifications.

**Revision:**

**Manuscript Section**

Page 1, Keywords

*HVDC cable, Insulation deterioration, Partial discharge, Protrusion defect, XLPE insulation*

**Specific comments 2:**

The "Introduction" section should be made much more impressive and enriched with references by adding and citing the trends in the area of the presented method.

**Answer:**

Thank you very much for your feedback. The writing in the introduction section of this article is indeed not attractive enough to readers, and the references cited are not sufficient. Now, the introduction has been revised to include research trends in the field.

**Revision:**

**Manuscript Section**

Page 2, Introduction

*The development of new energy technologies, such as photovoltaic power generation [1,2], has significantly increased the use of renewable energy in daily life [3-6] and its integration into the power grid [7]. This growth has sparked interest in flexible HVDC (High Voltage Direct Current) transmission, which can efficiently connect renewable energy sources to the grid. As the voltage level of HVDC lines continues to rise, the safety of critical power equipment, such as transformers [8] and HVDC cables, has become a growing concern. Among these, the HVDC cable is essential for transmission, but it is prone to failure due to internal aging processes.*

*To mitigate cable aging, two primary approaches are adopted: enhancing insulation materials and assessing the cable’s condition. Insulation performance can be improved by developing new materials [9,10] or modifying existing ones, like cross-linked polyethylene (XLPE), through techniques such as nano-doping [11-13]. On the other hand, evaluating the physical phenomena before aging faults occur can help predict cable failure. One such phenomenon is partial discharge (PD), which often results from defects in the insulation. Continuous PD accelerates aging, compromising the insulation’s integrity [14,15].*

*Cable defects can be broadly categorized into common types, including voids, cracks, water trees, and protrusions, each of which contributes differently to the formation and progression of PD. Voids are air gaps or pockets within the insulation material, which significantly disrupt the electric field and are often the starting point for PD. Cracks result from mechanical stresses and thermal cycling, leading to insulation breakdown. Water trees are a form of defect caused by moisture ingress, which accelerates insulation degradation under high-voltage conditions. Among these, protrusions are small, localized geometric imperfections that extend from the semi-conductive layer into the insulation. These protrusions distort the electric field, creating localized stress points that can trigger partial discharges.*

*Among insulation defects, protrusions are small, localized geometric imperfections that extend from the semi-conductive layer into the insulation. These protrusions distort the electric field, creating localized stress points that can trigger partial discharges. As PD continues, protrusions evolve, forming arborescent discharge channels that grow over time. Once the electric field within the remaining insulation exceeds the breakdown threshold, the discharge channel can rapidly propagate, leading to insulation failure. Therefore, understanding the formation and progression of these discharge channels is crucial for preventing cable breakdown.*

*Most studies on PD in XLPE cables under DC voltage focus on pin-plate electrode configurations, which are insufficient to model real-world cable defects [17,18]. Furthermore, the discharge channels are difficult to observe directly in situ, though PD characteristics remain strongly correlated with their development [19]. To address this, we investigate the behavior of protrusion defects in DC cables under controlled voltage and temperature conditions, monitoring the variation in PD characteristics. A finite element simulation model is established to simulate the electric field distribution and the development of discharge channels, based on electron avalanche theory. This model helps to explore the relationship between electric field distortion at the defect site, discharge magnitude, and channel size. The study aims to provide a new method for monitoring the stages of defect progression, offering insights into the early detection of faults in HVDC cables.*

*The results of this research have significant implications for the power transmission sector. Understanding early-stage cable degradation can improve the reliability of HVDC systems, particularly in high-stress environments such as offshore wind farms or remote solar installations. This approach also enhances predictive maintenance, reducing the risk of sudden cable failures and ensuring stable power grid operation.*

*Moreover, the methods developed in this research can be extended to other high-voltage equipment, such as transformers, circuit breakers, and capacitors, where PD also plays a critical role in aging and failure. Early detection of PD can thus contribute to improved safety and efficiency in the broader electrical infrastructure.*

**Specific comments 3:**

The paper's classification of context is not addressed at the end of the introduction.

**Answer:**

Thank you very much for your feedback. Considering your opinion, we have now added a classification of context for this article at the end of the introduction

**Revision:**

**Manuscript Section**

Page 2, Introduction

*The development of new energy technologies, such as photovoltaic power generation [1,2], has significantly increased the use of renewable energy in daily life [3-6] and its integration into the power grid [7]. This growth has sparked interest in flexible HVDC (High Voltage Direct Current) transmission, which can efficiently connect renewable energy sources to the grid. As the voltage level of HVDC lines continues to rise, the safety of critical power equipment, such as transformers [8] and HVDC cables, has become a growing concern. Among these, the HVDC cable is essential for transmission, but it is prone to failure due to internal aging processes.*

*To mitigate cable aging, two primary approaches are adopted: enhancing insulation materials and assessing the cable’s condition. Insulation performance can be improved by developing new materials [9,10] or modifying existing ones, like cross-linked polyethylene (XLPE), through techniques such as nano-doping [11-13]. On the other hand, evaluating the physical phenomena before aging faults occur can help predict cable failure. One such phenomenon is partial discharge (PD), which often results from defects in the insulation. Continuous PD accelerates aging, compromising the insulation’s integrity [14,15].*

*Among insulation defects, protrusions are small, localized geometric imperfections that extend from the semi-conductive layer into the insulation. These protrusions distort the electric field, creating localized stress points that can trigger partial discharges. As PD continues, protrusions evolve, forming arborescent discharge channels that grow over time. Once the electric field within the remaining insulation exceeds the breakdown threshold, the discharge channel can rapidly propagate, leading to insulation failure. Therefore, understanding the formation and progression of these discharge channels is crucial for preventing cable breakdown.*

*Most studies on PD in XLPE cables under DC voltage focus on pin-plate electrode configurations, which are insufficient to model real-world cable defects [17,18]. Furthermore, the discharge channels are difficult to observe directly in situ, though PD characteristics remain strongly correlated with their development [19]. To address this, we investigate the behavior of protrusion defects in DC cables under controlled voltage and temperature conditions, monitoring the variation in PD characteristics. A finite element simulation model is established to simulate the electric field distribution and the development of discharge channels, based on electron avalanche theory. This model helps to explore the relationship between electric field distortion at the defect site, discharge magnitude, and channel size. The study aims to provide a new method for monitoring the stages of defect progression, offering insights into the early detection of faults in HVDC cables.*

*The results of this research have significant implications for the power transmission sector. Understanding early-stage cable degradation can improve the reliability of HVDC systems, particularly in high-stress environments such as offshore wind farms or remote solar installations. This approach also enhances predictive maintenance, reducing the risk of sudden cable failures and ensuring stable power grid operation.*

*Moreover, the methods developed in this research can be extended to other high-voltage equipment, such as transformers, circuit breakers, and capacitors, where PD also plays a critical role in aging and failure. Early detection of PD can thus contribute to improved safety and efficiency in the broader electrical infrastructure.*

**Specific comments 4:**

In line 91, you mentioned “the total length of the cable is 45 cm," while it is in Fig. 2.a 25 cm.

**Answer:**

Thank you very much for reading this article in detail. The 25cm marked in the image corresponds to the length of the gray insulation layer on top of it. There are also 3cm stripped copper wires and 7cm insulation layer on both sides of the cable, so the total length of the cable is still 45cm.

**Specific comments 5:**

In line 103, you mentioned that “C voltage is rectified by a high-voltage silicon stack, filtered through a 0.1-μF stabilizing capacitor." There is a way of utilizing the line leakage resistance for better filtering. Moreover, the frequency range you mentioned in line 111 is a source of harmonics. Actually, the switching harmonics are a big issue, which you did not consider in the paper. So, this section should be made much more impressive and enriched with references by adding and citing the trends in the area of harmonics mitigation by passive power filters. E.g., High-frequency harmonics suppression in high-speed railway through magnetic integrated LLCL filter; Magnetic integrated double-trap filter utilizing the mutual inductance for reducing current harmonics in high-speed railway traction inverters; Magnetic integrated LLCL filter with resonant ‎frequency above Nyquist frequency; and Enhancing power quality of high-speed railway traction converters by fully integrated T-LCL filter. ‎ Some of these studies, which are published in the same journal, PLOS ONE, are recent and discuss the latest techniques.

**Answer:**

Thank you very much for your valuable feedback. After reading the literature you recommended in detail, I believe that this part of the content can help me deal with harmonic problems and suppress them effectively. Therefore, I have added the application of these literature in the original text to make the theory of this article more convincing

**Specific comments 6:**

There are a few writing issues. Also, the correct punctuation rules are not observed in the text.

**Answer:**

Thank you very much for your feedback. There were some inappropriate grammar and spelling issues during the writing process of this article. We have made modifications to the entire content to meet the requirements of this article.

**Specific comments 7:**

Many figures and equations were not mentioned in the main text or mentioned after they were presented. You need to mention them first.

**Answer:**

Thank you very much for your feedback. During the writing process of this article, it was originally a draft written in the format set by oneself. Therefore, for the convenience of typesetting, some images appeared before the references. Now, modifications have been made to address these situations.

**Revision:**

**Manuscript Section**

Page 7

*The schematic diagram of the two-dimensional geometric model is shown in Fig 4 (A), and the geometric dimensions are consistent with those in Table 1. Free triangular mesh is selected for grid division because the time scale of gas discharge migration is small and it is greatly affected by an electric field, it needs to be divided very finely to ensure that the charge migration process of the simulation process can be accurately completed. The defect cavity was calibrated to a hydrodynamic partition, the mesh size was "extremely fine", and the maximum cell size was set to 2 μm, containing a total of 48882 in-domain cells. The division diagram is shown in Fig 4 (B).*

|  |  |
| --- | --- |
| *(a) Geometric structure* | *(b) mesh generation* |

*Fig 4. Schematic diagram of discharge simulation and grid division.*

Page 8

*The entire discharge process is illustrated in the discharge spectrum shown in Fig 6. The discharge duration is divided into four stages: the first stage occurs during the discharge time from 0 to 40 minutes; the second stage takes place during the voltage application time from 41 to 110 minutes; the third stage occurs during the voltage application time from 111 to 140 minutes, and the fourth stage transpires during the voltage application time from 141 to 156 minutes. The basis of our discharge stage division is a sudden change in the trend of discharge frequency and discharge quantity.*

 *Fig 6. The discharge pattern of the whole experiment process of the protrusion defect.*

**Specific comments 8:**

You need to compare the ability of the proposed method with the literature to show what the benefit of the proposed technique is. Moreover, you have to mention the limitations of the proposed technique.

**Answer:**

We appreciate the reviewer’s valuable feedback. In response to the request for a comparison of the proposed method with existing literature and an acknowledgment of its limitations, we have updated the manuscript accordingly. The proposed technique focuses on partial discharge (PD) measurements to analyze the deterioration of XLPE cables with protrusion defects. This method stands out by not only monitoring PD but also categorizing the discharge characteristics into different stages, which enables a more detailed understanding of defect evolution. Previous studies have primarily focused on detecting PD in a general sense, without differentiating between the various stages of discharge. This limitation makes it harder to predict the progression of defects accurately. For example, [Di Fatta et al., 2024] utilized PD for assessing the condition of HVDC cables, but their approach did not provide an in-depth analysis of how discharge parameters evolve over time. In contrast, our study takes a more comprehensive approach by examining the trends in discharge frequency, quantity, and cumulative discharge, offering a better understanding of defect development. Furthermore, the simulation results in our study complement the experimental findings, adding credibility to the proposed method.

The key benefit of our technique lies in its stage-based analysis, which allows for a better prediction of defect progression and helps to identify when a defect is approaching a critical stage, something that traditional methods might miss. By analyzing changes in discharge frequency, quantity, and cumulative discharge, our method provides a more precise and early detection of cable insulation deterioration, surpassing the qualitative assessments typically seen in other approaches. Additionally, the integration of experimental and simulation data strengthens the reliability of our technique. However, there are limitations to the method. Its accuracy can be influenced by external factors like temperature, humidity, and the operating voltage of the cable, which may cause variations in PD characteristics. Although our experiments were conducted under controlled conditions (50°C and 80 kV), further research is necessary to evaluate the impact of different environmental factors. Moreover, the proposed method requires sophisticated equipment for PD detection and detailed data analysis, which could limit its practical application, especially in field environments where resources may be limited. Finally, while the method is optimized for protrusion defects in XLPE insulation, applying it to othaer defect types, such as air gaps or fouling defects, may require further adjustments to account for differences in discharge behavior. We believe that these additions provide a more comprehensive understanding of the strengths and limitations of the proposed technique.

[1]. Di Fatta, A., Romano, P., Rizzo, G., Ala, G., & Imburgia, A. (2024). The Interaction Between Electric Field and Partial Discharges Simultaneously Detected in a HVDC Cable Under Operating Conditions. *IEEE Access*, 12, 140171-140184.

**Specific comments 9:**

Could you insert any reference for equations? It is more suitable to determine the references or explain the design procedure that was adopted to get these equations.

**Answer:**

Thank you for your insightful comment. We have carefully reviewed the equations presented in the manuscript and made the necessary revisions to address your concerns. Specifically, references have been added to support the derivation and theoretical basis of the equations used in the Partial Discharge Simulation and Discussion sections. These citations clarify the origins of the hydrodynamic gas equations and the three-capacitance model, ensuring transparency regarding the methodologies adopted. For equations developed as part of our theoretical framework, we have provided additional explanations detailing the assumptions, modeling steps, and reasoning behind their formulation. These revisions highlight how the equations were derived based on prior studies and theoretical principles while demonstrating their adaptation to the specific conditions studied in this work. We appreciate the reviewer’s suggestion, which has significantly enhanced the rigor and clarity of our manuscript.

**Specific comments 10:**

The conclusion should be point-wise

**Answer:**

Thank you very much for your feedback on the conclusion section of this article. Based on your suggestions, I have conducted a more in-depth study and discussion on the conclusion section of this article by combining the following four aspects: Quantitative Analysis Validation of Simulation Results Impact of Other Defects Proactive Measures and Mitigation.

**Revision:**

**Manuscript Section**

Page 15, Conclusion

*This study investigates the evolution of partial discharge (PD) characteristics in cross-linked polyethylene (XLPE) cables with protrusion defects under long-term applied DC voltage. The results highlight the significant role of partial discharge in deteriorating the insulation of XLPE cables, thereby reducing their service life. By analyzing the evolution of discharge quantity and amplitude, we can infer the development of defects within the cable insulation. The findings suggest that the insulation performance remains stable when discharge amplitude shows little variation. However, a rapid increase in cumulative discharge and discharge amplitude indicates the progression of the protrusion defect, particularly in the fourth stage of discharge, which marks a critical point of failure risk.*

*A more rigorous, quantitative assessment of partial discharge characteristics reveals that when both cumulative discharge and discharge amplitude rise significantly, the cable is likely to be approaching failure. Future studies could focus on establishing numerical thresholds for these changes to provide clearer diagnostic markers for cable health. Additionally, further simulation and experimental validation are necessary to refine these predictive models and ensure their applicability under various operational conditions.*

*The insights gained from this study also open the door for extending the analysis to other types of defects, such as air gap and fouling defects, which may have different discharge behaviors. The interaction of these defects with the electric field in the insulation could alter the discharge dynamics, and understanding this relationship is crucial for improving diagnostic accuracy and extending cable lifespan.*

*Furthermore, the study underscores the importance of early detection techniques for partial discharge and suggests the development of more advanced monitoring systems that could detect subtle changes in discharge characteristics before catastrophic failure occurs. The integration of these diagnostic tools could play a key role in enhancing the maintenance and reliability of high-voltage direct current (HVDC) cable systems, especially in critical infrastructure applications.*

*This paper demonstrates that partial discharge analysis is a valuable tool for assessing the condition of XLPE cables with protrusion defects. Further work in this field should aim to refine defect models, enhance diagnostic methodologies, and explore mitigation strategies to prevent cable damage, thereby improving the safety and longevity of HVDC cable networks.*

**Specific comments 11:**

In the technical writing, it is not true to write “we, I, our, etc.”.

**Answer:**

Thank you very much for your valuable feedback. In the process of writing the article, there was an overly colloquial expression that made it less rigorous. We have now made modifications to these subjects to make the writing style both formal and in line with the norms of academic papers

**Revision:**

**Manuscript Section**

Page 6, line 171

*Although the degree of electric field distortion at the defect has been understood, it is the partial discharge at the defect that really damages the insulation material. Partial discharge is a complex and constantly changing physical process.*

Page 8, line 254

*The entire discharge process is illustrated in the discharge spectrum shown in Fig. 6. The discharge duration is divided into four stages: the first stage occurs during the discharge time from 0 to 40 minutes; the second stage takes place during the voltage application time from 41 to 110 minutes; the third stage occurs during the voltage application time from 111 to 140 minutes; and the fourth stage transpires during the voltage application time from 141 to 156 minutes. The basis for the division of discharge stages is a sudden change in the trend of discharge frequency and discharge quantity.*

Page 18, line 467

*By analyzing the evolution of discharge quantity and amplitude, we can infer the development of defects within the cable insulation. The findings suggest that the insulation performance remains stable when discharge amplitude shows little variation. However, a rapid increase in cumulative discharge and discharge amplitude indicates the progression of the protrusion defect, particularly in the fourth stage of discharge, which marks a critical point of failure risk.*

**Specific comments 12:**

Some of the references are old. A state-of-the-art (SotA) review is preferred to show the real advantages of the proposed method against the recent literature. All the references have no DOI. Many references are conference papers.

**Answer:**

Thank you very much for your constructive feedback. I appreciate your suggestion to include more recent references and provide a state-of-the-art (SotA) review to better highlight the advantages of the proposed method in comparison to the latest literature. In response, I have updated the references section by including more recent papers and a comprehensive review of the current state-of-the-art techniques in the field. This should help to clarify the novelty and significance of the proposed approach.

Regarding the references without DOIs and the inclusion of conference papers, I have revised the citations to ensure that all references are properly formatted with DOIs where available. Additionally, I have replaced some of the conference papers with peer-reviewed journal articles to strengthen the academic rigor of the manuscript.

Thank you once again for your valuable insights. I believe these revisions have enhanced the quality of the manuscript.
