# Supplementary material for: Analysis of the deterioration process of DC XLPE cable with protrusion defect based on the development of partial discharge
Source: PLoS One. 2025 Jun 25;20(6):e0326271. doi: 10.1371/journal.pone.0326271 (PMC12192135; doi:10.1371/journal.pone.0326271)
Supplement: Attachment — Submitted filename: Response_to_Reviewers_auresp_2.docx [file pone.0326271.s005.docx]

**Revision Report for Manuscript** **PONE-D-24-49770R1**

**Dear Editors:**

We gratefully appreciate both editors and reviewers for their careful reading, helpful comments, and constructive suggestions, which have significantly improved the quality of our manuscript entitled “**Analysis of the deterioration process of DC XLPE cable with** **protrusion defect based on the development of partial discharge**” (**Manuscript Number: PONE-D-24-49770R1**).

We have carefully considered all comments from the reviewers and revised our manuscript accordingly. In the following section, we summarize our responses to each comment from the reviewers. We believe that our responses have well addressed all concerns from the reviewers. Here, we would like to re-submit the revised manuscript to *PLOS ONE*. Hope these revisions could present a better view to the readers and make the paper acceptable for publication in the journal.

Looking forward to hearing from you soon. Have a nice day.

Best regards,

The Authors

Shanghai Jiao Tong University,

Shanghai, 200240, China

+86-18915198870

z1mubaiyu@sjtu.edu.cn

**Responses to Additional Editor Comments:**

Thank you for your feedback and for acknowledging the revisions made in response to the previous comments. We appreciate the reviewer’s suggestions and understand the need for further revisions. We will carefully address the remaining comments and ensure that all requested changes are made thoroughly, so as to avoid any delays in the next revision.

Thank you again for your time and valuable input.

**Responses to Reviewer #1:**

Thank you very much for your thoughtful review and feedback. We are grateful for your positive assessment, and we are pleased that the revisions addressed your comments to your satisfaction. We appreciate your support and are glad the paper meets the standards for publication.

Thank you again for your time and valuable input.

**Response to Reviewer #2:**

**General comments:**

First of all, I appreciate the authors for the perfect responses and improvements. I am delighted to inform you that this manuscript is very strong now, and I believe that this paper will gain a high number of citations in the future. The authors made a major correction from the previous round. However, this paper requires small corrections because some corrections from the previous round were not done. Kindly find the following notes for further improvements:

**Answer:**

Thank you very much for your positive feedback and kind words regarding our revisions. We are grateful for your continued support and thoughtful comments, which have significantly contributed to strengthening the manuscript. We acknowledge the small corrections you have pointed out, and we sincerely apologize for the oversight in implementing some of the previous revisions. We will carefully address the remaining comments and make the necessary adjustments to ensure the paper meets the required standards.

We appreciate your time and effort in reviewing the manuscript and look forward to submitting the final version with all corrections made.

We modify the manuscript with a clearer expression and show the changes made in the revised paper in red color. The specific comments were answered point by point as follows.

**Specific comments 1:**

In the previous round, comment 3 was “The paper's classification of context is not addressed at the end of the introduction.” You have replied to this comment clearly and showed the revision that you have done in the revised manuscript. What I meant is that it is known that at the end of the introduction to present the outline of the paper, like this example: “The rest of this paper is organized as follows. Section 2 would first show the... The... are presented in Section 3. Section 4 provides... Finally, this paper is concluded in Section 5.” So, you may consider this way.

**Answer:**

Thank you for your insightful comment regarding the classification of the paper's structure at the end of the introduction. In response to your suggestion, we have revised the manuscript to include a clear outline of the paper's organization. We have now added the following section to the conclusion of the introduction:

"The rest of this paper is organized as follows: Section 2 presents the experimental setup and methodology used to investigate partial discharge behavior in XLPE cables. Section 3 details the results of the partial discharge experiments, analyzing the stages of discharge and their implications for cable deterioration. Section 4 discusses the findings in the context of insulation degradation mechanisms, offering a comparison to existing literature. Finally, Section 5 concludes the paper, summarizing the key insights and suggesting future directions for HVDC cable design and testing."

We hope this revision adequately addresses your comment.

**Revision:**

**Manuscript Section**

Page 2, Introduction

*The rest of this paper is organized as follows: Section 2 presents the experimental setup and methodology used to investigate partial discharge behavior in XLPE cables. Section 3 details the results of the partial discharge experiments, analyzing the stages of discharge and their implications for cable deterioration. Section 4 discusses the findings in the context of insulation degradation mechanisms, offering a comparison to existing literature. Finally, Section 5 concludes the paper, summarizing the key insights and suggesting future directions for HVDC cable design and testing.*

**Specific comments 2:**

In the previous round, comment 8 was, “You need to compare the ability of the proposed method with the literature to show what the benefit of the proposed technique is. Moreover, you have to mention the limitations of the proposed technique.” You have replied to this comment clearly. However, you did not show the revision that you have done in the revised manuscript.

**Answer:**

Thank you for your valuable feedback regarding the comparison with the literature and the limitations of the proposed method. In response to your comment, we have revised the manuscript to include a more explicit comparison of the proposed method with existing methods. Specifically, in the 2.4 section, we now highlight the advantages of the proposed technique over previous approaches and provide a critical evaluation of its limitations.

We believe this addition enhances the clarity of the benefits of the proposed method and acknowledges its boundaries, as requested.

**Revision:**

**Section 2.4**

Page 7

*The generation and disappearance of charged particles are described by the equation of source term (5~7). In the above equation, the specific values of the above simulation parameters were obtained from the literature[27,28].*

**Section 2.4**

Page 8

*To provide a clearer understanding of the logical flow of this article, a flowchart is presented below. This flowchart summarizes the overall logical structure and progression of the content: it begins with the introduction section, which outlines the research background and issues, as well as clarifies the research objectives; next, the methods section elaborates on the research design and experimental approaches; then, the results section presents the analyzed data and key findings; following that, the discussion section interprets the significance of the research results and compares them with existing literature; finally, the conclusion section summarizes the research contributions and offers prospects for future work.*

**Specific comments 3:**

In the previous round, comment 11 was, “In the technical writing, it is not true to write “we, I, our, etc.”.” You have replied to this comment clearly and showed the revision that you have done in the revised manuscript. However, there are still some places that were not corrected, or some places in the new content were not written considering this comment.

**Answer:**

Thank you for your comment regarding the use of first-person pronouns in the manuscript. We have carefully reviewed the document and made revisions to remove the instances where "we", "I", and "our" were used, in accordance with your suggestion. The necessary changes have been made to ensure the manuscript follows appropriate technical writing standards.

We hope this revision satisfies your concerns.

**Revision:**

**Introduction Section**

Page 2

*Most studies on PD in XLPE cables under DC voltage focus on pin-plate electrode configurations, which are insufficient to model real-world cable defects [17,18]. Furthermore, the discharge channels are difficult to observe directly in situ, though PD characteristics remain strongly correlated with their development [19]. To address this, by investigating the behavior of protrusion defects in DC cables under controlled voltage and temperature conditions, monitoring the variation in PD characteristics. A finite element simulation model is established to simulate the electric field distribution and the development of discharge channels, based on electron avalanche theory. This model helps to explore the relationship between electric field distortion at the defect site, discharge magnitude, and channel size. The study aims to provide a new method for monitoring the stages of defect progression, offering insights into the early detection of faults in HVDC cables.*

**Section 3.1**

Page 9

*The entire discharge process is illustrated in the discharge spectrum shown in Fig 6. The discharge duration is divided into four stages: the first stage occurs during the discharge time from 0 to 40 minutes; the second stage takes place during the voltage application time from 41 to 110 minutes; the third stage occurs during the voltage application time from 111 to 140 minutes, and the fourth stage transpires during the voltage application time from 141 to 156 minutes. The basis of the discharge stage division is a sudden change in the trend of discharge frequency and discharge quantity.*

**Conclusion Section**

Page 17

*This study investigates the evolution of partial discharge (PD) characteristics in cross-linked polyethylene (XLPE) cables with protrusion defects under long-term applied DC voltage. The results highlight the significant role of partial discharge in deteriorating the insulation of XLPE cables, thereby reducing their service life. By analyzing the evolution of discharge quantity and amplitude,* *it can be inferred that the development of defects within the cable insulation. The findings suggest that the insulation performance remains stable when discharge amplitude shows little variation. However, a rapid increase in cumulative discharge and discharge amplitude indicates the progression of the protrusion defect, particularly in the fourth stage of discharge, which marks a critical point of failure risk.* *The entire discharge process is illustrated in the discharge spectrum shown in Fig 6. The discharge duration is divided into four stages: the first stage occurs during the discharge time from 0 to 40 minutes; the second stage takes place during the voltage application time from 41 to 110 minutes; the third stage occurs during the voltage application time from 111 to 140 minutes, and the fourth stage transpires during the voltage application time from 141 to 156 minutes. The basis of our discharge stage division is a sudden change in the trend of discharge frequency and discharge quantity.*
