# Supplementary material for: Analysis of the deterioration process of DC XLPE cable with protrusion defect based on the development of partial discharge
Source: PLoS One. 2025 Jun 25;20(6):e0326271. doi: 10.1371/journal.pone.0326271 (PMC12192135; doi:10.1371/journal.pone.0326271)
Supplement: Attachment — Submitted filename: PONE-D-24-49770_R2_reviewer Comments.docx [file pone.0326271.s004.docx]

The authors made a major correction from the previous round. However, this paper requires small corrections because some corrections from the previous round were not done. Kindly find the following notes for further improvements:

1. In the previous round, comment 2 was, (In the previous round, comment 8 was, “You need to compare the ability of the proposed method with the literature to show what the benefit of the proposed technique is. Moreover, you have to mention the limitations of the proposed technique.” You have replied to this comment clearly. However, you did not show the revision that you have done in the revised manuscript.). You added this paragraph in **Section 2.4**, Page 8 “To provide a clearer understanding of the logical flow of this article, a flowchart is presented below. This flowchart summarizes the overall logical structure and progression of the content: it begins with the introduction section, which outlines the research background and issues, as well as clarifies the research objectives; next, the methods section elaborates on the research design and experimental approaches; then, the results section presents the analyzed data and key findings; following that, the discussion section interprets the significance of the research results and compares them with existing literature; finally, the conclusion section summarizes the research contributions and offers prospects for future work.”, which is a repeat of the paper's classification of context at the end of the introduction, not the required correction. You need to reconsider this comment.
2. In the previous round, comment 3 was, (“In the technical writing, it is not true to write “we, I, our, etc.”.” You have replied to this comment clearly and showed the revision that you have done in the revised manuscript. However, there are still some places that were not corrected, or some places in the new content were not written considering this comment.) You made some modifications but some of them are not correct. Specifically, in **Introduction Section**, Page 2 “To address this, by investigating the behavior of protrusion defects in DC cables under controlled voltage and temperature conditions, monitoring the variation in PD characteristics.” And in **Conclusion Section**, Page 17, “By analyzing the evolution of discharge quantity and amplitude, it can be inferred that the development of defects within the cable insulation.” These two sentences are not grammatically true.

The revised manuscript can be accepted for publication if these two minor notices were considered in the final version. The paper is not required to be sent again to the reviewer. You just consider these notes before publishing the paper.
