# Supplementary material for: Analysis of the deterioration process of DC XLPE cable with protrusion defect based on the development of partial discharge
Source: PLoS One. 2025 Jun 25;20(6):e0326271. doi: 10.1371/journal.pone.0326271 (PMC12192135; doi:10.1371/journal.pone.0326271)
Supplement: Attachment — Submitted filename: Response_to_Reviewers_auresp_3.docx [file pone.0326271.s006.docx]

**Revision Report for Manuscript** **PONE-D-24-49770R2**

**Dear Editors:**

We gratefully appreciate both editors and reviewers for their careful reading, helpful comments, and constructive suggestions, which have significantly improved the quality of our manuscript entitled “**Analysis of the deterioration process of DC XLPE cable with** **protrusion defect based on the development of partial discharge**” (**Manuscript Number: PONE-D-24-49770R2**).

We have carefully considered all comments from the reviewers and revised our manuscript accordingly. In the following section, we summarize our responses to each comment from the reviewers. We believe that our responses have well addressed all concerns from the reviewers. Here, we would like to re-submit the revised manuscript to *PLOS ONE*. Hope these revisions could present a better view to the readers and make the paper acceptable for publication in the journal.

Looking forward to hearing from you soon. Have a nice day.

Best regards,

The Authors

Shanghai Jiao Tong University,

Shanghai, 200240, China

+86-18915198870

z1mubaiyu@sjtu.edu.cn

**Response to Reviewer #2:**

**Specific comments 1:**

1. In the previous round, comment 2 was, (In the previous round, comment 8 was, “You need to compare the ability of the proposed method with the literature to show what the benefit of the proposed technique is. Moreover, you have to mention the limitations of the proposed technique.” You have replied to this comment clearly. However, you did not show the revision that you have done in the revised manuscript.). You added this paragraph in Section 2.4, Page 8 “To provide a clearer understanding of the logical flow of this article, a flowchart is presented below. This flowchart summarizes the overall logical structure and progression of the content: it begins with the introduction section, which outlines the research background and issues, as well as clarifies the research objectives; next, the methods section elaborates on the research design and experimental approaches; then, the results section presents the analyzed data and key findings; following that, the discussion section interprets the significance of the research results and compares them with existing literature; finally, the conclusion section summarizes the research contributions and offers prospects for future work.”, which is a repeat of the paper's classification of context at the end of the introduction, not the required correction. You need to reconsider this comment.

**Answer:**

We sincerely thank the reviewer for this important reminder. In the previous revision, we inadvertently misunderstood the intent of the comment and added only a general flowchart in Section 2.4, which repeated the structural overview from the Introduction and did not constitute a valid response.

In this revised version, we have now explicitly added a new subsection titled **“4.4 Comparison with existing literature and method limitations”** (page 17), in which we:

Compare our method (cable-based protrusion defect model with combined experiment and simulation) with traditional approaches (needle-plate models),

Highlight its advantages in representing realistic field scenarios and capturing both macroscopic and microscopic discharge behaviors,

Acknowledge limitations regarding thermal condition control, lack of cyclic voltage or mechanical stress testing, and simplifications in the simulation model.

We hope this revision fully addresses the reviewer’s concern.

**Revision:**

**Manuscript Section**

Page 17

**4.4 Comparison with existing literature and method limitations**

*Compared to conventional needle-plate electrode models used in studies of DC electrical treeing in XLPE insulation, the method proposed in this paper uses a cable structure with real protrusion defects to better simulate field operation scenarios. This experimental configuration allows for more realistic electric field distributions and partial discharge paths, as seen in ^[14, 17–19]^. While needle-plate models are convenient for reproducible laboratory testing, they often fail to reflect the influence of cable geometry, semiconductive shielding, and defect material characteristics on discharge evolution.*

*Additionally, the combination of experimental discharge data and hydrodynamic simulations based on electron avalanche theory provides insight into the microscopic discharge process and its correlation with macroscopic discharge characteristics, which is not commonly addressed in literature ^[25–27]^. This multi-physics modeling approach offers a more comprehensive understanding of insulation degradation, beyond the phenomenological analysis found in many previous studies.*

*However, this study also has limitations. The temperature conditions were fixed at 50 °C, which does not fully reflect the dynamic thermal variations experienced by cables in service. Moreover, the discharge behavior under long-term cyclic voltage application or mechanical stress was not explored. From a modeling perspective, the assumptions in the plasma simulation (e.g., neglecting space charge accumulation or surface charge relaxation dynamics) may limit its applicability under certain field conditions. Future work should address these factors and validate the results under broader operating conditions.*

**Specific comments 2:**

2. In the previous round, comment 3 was, (“In the technical writing, it is not true to write “we, I, our, etc.”.” You have replied to this comment clearly and showed the revision that you have done in the revised manuscript. However, there are still some places that were not corrected, or some places in the new content were not written considering this comment.) You made some modifications but some of them are not correct. Specifically, in Introduction Section, Page 2 “To address this, by investigating the behavior of protrusion defects in DC cables under controlled voltage and temperature conditions, monitoring the variation in PD characteristics.” And in Conclusion Section, Page 17, “By analyzing the evolution of discharge quantity and amplitude, it can be inferred that the development of defects within the cable insulation.” These two sentences are not grammatically true.
The revised manuscript can be accepted for publication if these two minor notices were considered in the final version. The paper is not required to be sent again to the reviewer. You just consider these notes before publishing the paper.

**Answer:**

We thank the reviewer for carefully identifying these remaining issues. In the current revision, we have corrected the grammatical errors and ensured alignment with academic writing standards. The specific changes are as follows:

**Revision:**

**Section 1**

Page 2

*Most studies on PD in XLPE cables under DC voltage focus on pin-plate electrode configurations, which are insufficient to model real-world cable defects [17,18]. Furthermore, the discharge channels are difficult to observe directly in situ, though PD characteristics remain strongly correlated with their development [19]. To address this issue, the behavior of protrusion defects in DC cables was investigated under controlled voltage and temperature conditions, and the variation in partial discharge (PD) characteristics was monitored. A finite element simulation model is established to simulate the electric field distribution and the development of discharge channels, based on electron avalanche theory. This model helps to explore the relationship between electric field distortion at the defect site, discharge magnitude, and channel size. The study aims to provide a new method for monitoring the stages of defect progression, offering insights into the early detection of faults in HVDC cables.*

**Section 5**

Page 18

*This study investigates the evolution of partial discharge (PD) characteristics in cross-linked polyethylene (XLPE) cables with protrusion defects under long-term applied DC voltage. The results highlight the significant role of partial discharge in deteriorating the insulation of XLPE cables, thereby reducing their service life.* *By analyzing the evolution of discharge quantity and amplitude, the development of defects within the cable insulation can be inferred. The findings suggest that the insulation performance remains stable when discharge amplitude shows little variation. However, a rapid increase in cumulative discharge and discharge amplitude indicates the progression of the protrusion defect, particularly in the fourth stage of discharge, which marks a critical point of failure risk.*
